# On the absence of the Green-tailed Trainbearer *Lesbia nuna* (Trochilidae) from Venezuela: an analysis based on environmental niche modelling

**DOI:** 10.3897/BDJ.5.e22092

**Published:** 2017-12-15

**Authors:** Paolo Ramoni Perazzi, Karl L. Schuchmann, Magdiel Ablan Bortone, Alejandra Soto Werschitz

**Affiliations:** 1 Centro de Simulación y Modelos, Facultad de Ingenieria and Laboratorio de Zoología Aplicada, Facultad de Ciencias, Universidad de Los Andes, Mérida, Venezuela; 2 Zoological Research Museum A. Koenig - ZFMK/Ornithology, Adenauerallee 160, 53113, Bonn, Germany; 3 Universidade Federal de Mato Grosso - UFMT/ Biociencias, Zoologia (Prof. Visitante), Member of the UFMT, Computational Bioacoustics Research Unit - CO.BRA, Cuiaba, Brazil; 4 Centro de Simulación y Modelos, Facultad de Ingenieria, Universidad de Los Andes,, Mérida, Venezuela; 5 Laboratorio de Zoología Aplicada, Facultad de Ciencias, Universidad de Los Andes, Mérida, Venezuela

**Keywords:** *Lesbia nuna gouldii*, suitable habitat, model, distribution, Colombia, Venezuela

## Abstract

**Background:**

*Lesbia nuna*, a hummingbird distributed in the tropical Andes, has been included in Venezuela's bird list on the basis of a specimen collected in 1873 at Sierra Nevada, Mérida and deposited in the Natural History Museum, London, with no further records for this country since then. This record, largely considered as valid by most authors, has been questioned by others, although without formal analyses.

**New information:**

The potential habitat range of the Green-Tailed Trainbearer, *Lesbia nuna gouldii* (Trochilidae), in the northern Andes from Ecuador to Venezuela was modelled, using maximum entropy niche modelling, environmental covariates and records from locations across the Colombian Andes. The predicted suitable habitat range corresponded well to the known range of the subspecies *L. n. gouldii* in Colombia and clearly excluded Sierra Nevada. Therefore, these analyses suggest that this species should be removed from the Venezuelan bird list.

## Introduction

*Lesbia nuna*, a hummingbird inhabiting the tropical Andes between 1700 and 3800 m (Schuchmann 1999), is currently divided into five (Clements et al. 2016) or seven (Züchner et al. 2017) subspecies, with *L. n. gouldii* being patchily distributed in the central and southern Andes of Colombia. There is also a specimen of this subspecies deposited in the Natural History Museum, London: a male labelled as collected by Anton Goering in 1873 at “Sierra Nevada, Merida, Venezuela”, with no further records made for this country since then (Restall et al. 2006). This record has been largely considered as valid by several authors (Meyer de Schauensee 1964, Meyer de Schauensee and Phelps 1978, Meyer de Schauensee 1982, Hilty and Brown 1986, Fjeldså and Krabbe 1990, Schuchmann 1999, Weller and Schuchmann 2004, Restall et al. 2006, Clements et al. 2016, Züchner et al. 2017), but questioned by Hilty (2003) and excluded by BirdLife International (2017), although without formal analyses.

Habitat alteration often follows the assessment of newly explored areas, with the concomitant extinctions (Fuller 2001). In such cases, species or populations from these areas known only from old single records are usually viewed with scepticism because the lack of further information suggests simply mislabelled specimens (for example Zimmer 1951). Some of these “mysteries” are rediscovered decades or centuries after their scientific descriptions (Scheffers et al. 2011), thus clarifying their situation. Nevertheless, most cases remain obscure, even after intensive and extensive field efforts, because the basic paradox of extinction documentation is that absence of evidence does not mean evidence of absence (Stine and Wagner 2005).

In such cases, ecological niche models (hereafter ENMs; Araújo and Peterson 2012, Peterson et al. 2012, Warren 2012) may constitute fast, convenient and reliable tools, since they can determine the actual distribution of secretive, restricted or rare taxa, information that can otherwise be challenging to obtain because robust empirical field information is often prohibitively expensive. These computer-based mathematical procedures approximate the areas containing a combination of ecological and environmental factors that may underpin the successful performance of a given taxon. ENMs have found extensive application in areas such as biogeography and phylogeography (Alvarado-Serrano and Knowles 2013, Catullo et al. 2013), or evolutionary biology (Lira-Noriega and Peterson 2014, Silva et al. 2014, Zhang et al. 2014) amongst others, being widely used to extrapolate observed patterns and predict taxa’s geographical distributions using occurrence information data and spatial layers of abiotic conditions (elevation, climate etc.) under past, current or future conditions. For example, Brown et al. (2015) successfully predicted new locality records for the Blue-fronted Lancebill (*Doryfera johannae*) from southeast Peru, *ca.* 470 km to the southeast of the range reported in literature.

The goal of the present study was to develop ENMs for the *L. n. gouldii* hummingbird subspecies in the northern Andes, from Ecuador to Venezuela, in order to determine whether its range actually reaches the Sierra Nevada in the latter country.

## Materials and methods

### Study area

The northern Andes from Ecuador to Venezuela (*ca.* 4°S to 11°N; sensu Montgomery et al. 2001) are characterised by their SW-NE trending resulting from the collision of the Panamanian arc and the Caribbean Plate against the South America plate (Kennan 2000) and the presence of parallel ridges. In Ecuador, however, the Western and Central Cordilleras are separated by a depression filled by sediments and the product of volcanic eruptions (Coltorti and Ollier 2000), while in Colombia both are separated by the deep intermontane Cauca Valley and the Magdalena valley separates the Central and Eastern Cordilleras. In Venezuela, the Eastern Cordillera splits into the Perijá and Mérida ranges.

In the northern Andes, climate is influenced by the Intertropical Convergence Zone, thus both slopes receive high annual precipitation rates (Jimenez and Oliver 2005) and the concomitant fluvial erosion maintains narrow mountain ranges compared to the remnant chain (Montgomery et al. 2001). In general, precipitation follows the unimodal rainfall pattern typical of Northern South America (Hastenrath 1984) or the typical bimodal pattern towards the Caribbean region (Taylor and Alfaro 2005).

The vegetation is distributed in longitudinal belts along slopes, imperfectly correlated with elevation. Upper slopes, below the snowline, are covered by "páramo", a herbaceous vegetation co-dominated by grasses and *Espeletia* (Compositae), while lower slopes are covered by forests, with a treeline of usually around 3200-3500 m associated with the 6°C isotherm of mean annual temperature (Holdridge 1967, van der Hammen 1974).

During the late Tertiary and the Quaternary, orbital forcing promoted several alternating cold and warm stages with major consequences for biota composition and distribution, especially during the Last Glacial Maximum (hereafter LGM, *ca.* 20 kyr BP), through mechanisms that are still being debated (Ramírez-Barahona and Eguiarte 2013). During LGM, vegetation belts were located at elevations 1000–1500 m below their current levels (van der Hammen 1974, Bush et al. 1990, Hooghiemstra and der Hammen 2004, Brunschön and Behling 2009, Valencia et al. 2010). This downslope disposition of belt vegetation types would have promoted the connectivity amongst currently isolated high-elevation ecological islands.

Today, the biota is also laterally segmented in physiognomically similar but taxonomically differentiable ecoregions occupying contiguous areas of the slopes (Olson et al. 2001). Such patterns, originating in the relictuation/expansion process of the ice ages, may be currently maintained not only by orographic barriers but also by ecological factors, such as edaphic differences similar to those thought to determine biodiversity patterns in the Amazon basin (Salo 1987).

García-Moreno and Fjeldså (2000) stated that “vicariant forms are valid species which remain ecologically incompatible for long periods, possibly because they are subject to uniform selection pressures”. Thus, considering the lack of evident physical barriers separating the parapatric taxa *L. n. gouldii and L. n. gracilis* as a control to correctly access the possibility of the occurrence of *L. nuna* in Venezuela, those ENMs that predicted the minimum area of suitable habitat for *L. n. gouldii* in Ecuador were selected.

### Spatial autocorrelation

The correction of biases in geographic space is an important step in avoidingmodels “overfitting” in environmental space. This is effected by sub-sampling the occurrence database and reducing the autocorrelation, sacrificing statistical power in favour of increasing the statistical independence of sampling units (Boria et al. 2014, de Oliveira et al. 2014, Varela et al. 2014). Therefore, ~10% (four) of the localities were removed from the dataset, selected through a recursive jackknife process that identified and progressively removed the localities with the highest contribution to autocorrelation based on Moran’s I coefficients using the “APE” package in R (Paradis et al. 2004, Paradis 2012) and a matrix of spatial weights (*W*_ij_) calculated as the inverse distance between locations *i* and *j* (1/*d*_ij_) which was estimated using the Haversine function in the “geosphere” package, version 1.3-13, in R (Hijmans 2015), for all non-categorical environmental variables using the values measured at the *L. nuna* occurrence points in Colombia.

### Environmental covariates

The bioclimatic and elevation layers provided by Worldclim, version 1.4 (Hijmans et al. 2005; from www.worldclim.com; 2.5 min resolution) were used. Two categorical covariates in the modelling process were also evaluated. First, geological provinces, based on geological data from Schenk et al. 1999, were coded. Second, soil types based on the Harmonised World Soil Database, version 1.2 (FAO et al. 2012) were coded. Although land-cover data has been successfully used to obtain more accurate spatial predictions (Tingley and Herman 2009), this covariate was excluded because the present dataset included specimens recorded/collected over a long period in a region characterised by high habitat transformation rates (Food and Agriculture Organization of the United Nations 2010).

The grids of variables used in this study were processed using the libraries “raster” version 2.3-12 (Hijmans 2014), “SDMTools” version 1.1-221 (VanDerWal et al. 2014), “maptools” version 0.8-30 (Bivand and Lewin-Koh 2014) and “rgdal” version 0.9-1 (Bivand et al. 2014), projected to the same coordinate system (EPSG:4248).

To remove the effects of multi-collinearity, Pearson's correlations between continuous variables, polyserial correlations between continuous and categorical variables and polychoric correlations between the categorical variables for those pixels where *L. nuna* was present, were tested. The highly correlated variables (r ≥ 0.75, p ≥ 0.001) were excluded from further analysis.

### Distribution predictions

Four variable combinations were evaluated: continuous variables alone (= climate and elevation; hereafter CON), continuous variables and geology (C+G), continuous variables and soil (C+S), and the combination of all (ALL). There are several ENM algorithms whose performances have been compared by several authors (e.g. Elith et al. 2006, Pearson et al. 2006, Phillips et al. 2006, Terribile et al. 2010, Poor et al. 2012, Merow and Silander 2014). However, only MaxEnt was chosen (version 3.3.3k, from http://biodiversityinformatics.amnh.org/open_source/maxent/, Phillips et al. 2004, Phillips et al. 2006) because the authors were not focused on model comparison since comparative studies demonstrated a similar or better performance forMaxEnt.

To remove the uncertainty that arises from differing use of pseudo-absence points, model predictions were cross-validated, conducting 10 runs, splitting training and test data on a 90:10 ratio and 1000 maximum iterations. As it was assumed that the *L. n. gouldii* occurrence data is biased, a bias grid was built with the same dimensions, cell size and projection as the environmental variables with relative sampling probabilities of 1 for the elevation range from 700 m a.s.l (the alleged lowest elevation range of this hummingbird during LGM) and above and -9999 elsewhere. Projections were made for each run and the average projection, according to the corresponding standard deviation, were created. The significance of each variable was tested through jackknifing.

Binary maps of presence/absence were created to facilitate the visualisation of model predictions. Different threshold methods result in discrepancies in omission errors and unsuitable areas (Nenzén and Araújo 2011, Bean et al. 2012, Liu et al. 2013, Syfert et al. 2013, Norris 2014). Therefore, the results obtained were compared for each of the four variable combinations using seven of the threshold methods available in MaxEnt: Minimum training presence, Fixed cumulative value 1, Fixed cumulative value 5, Fixed cumulative value 10, 10 percentile training presence, Equal training sensitivity and specificity and Maximum training sensitivity plus specificity.

In each case, the mean logistic threshold value was used from the ten runs to determine the amount of false negatives (omissions) and the suitable area predicted for the control country (Ecuador), selecting those having the lower false negatives (omissions) and, at the same time, predicting the smaller area for the control country to determine the suitable area in Venezuela.

## Data resources

### Occurrence records

For ENMs, information was gathered on presence localities for *L. nuna* in Colombia which was considered as belonging to *L. n. gouldii* from three main sources. First, information relating to 21 collections from Canada, Colombia, The Netherlands, United Kingdom and United States, whose information is provided online in VertNet (vertnet.org), BioMap (www.biomap.net), or gbif.org (2017) or was kindly provided by the respective curator after request was gathered. Second, information from specialised articles on fauna inventories or bird ecology (Willis and Schuchmann 1993, Olivares 1996, Losada-Prado et al. 2005, Echeverry-Galvis and Morales-Rozo 2007, Agudelo-Álvarez et al. 2010, Amaya-Villarreal and Renjifo 2010, Peraza 2011, Andrade-L and Benitez-Castañeda 2012) was obtained. Third, field recordings and videos reported in the specialised databases xeno-canto (www.xeno-canto.org) and the internet bird collection www.hbw.com/ibc) were obtained. Duplicate and redundant localities were removed from the dataset. Records of dubious origin and outliers with respect to the published geographic range were also excluded from the analyses.

The information provided by the citizen-science data was used, i.e. human observations (hereafter Appendix 2, available at http://cobra.ic.ufmt.br/web/guest/publications-data_sets) from the web-based repositories eBird Basic Dataset (2017), (Sullivan et al. 2009) and gbif.org (2017), wereexcluded from ENMs but considered as an additional control against which to compare the predictions generated.

The quality of georeferencing of these localities varied from GPS recordings to coordinates of the nearest towns listed on specimen labels. A variety of gazetteers and scientific publications to infer coordinates from the available locality information or to corroborate/correct this information when provided by the source consulted were used.

There is a specimen of *L. nuna* in the American Museum of Natural History (No. 38126) whose locality is simply “Pamplona” and this isprobably the reason why authors such as Meyer de Schauensee (1982) included Norte de Santander Department, Colombia, within the range of *L. nuna*. That is outside the remaining collecting localities: a “very uncertain locality” according to BioMap. Therefore, two sets of analyses were undertaken: one including and another excluding such a record. The rationale behind this was (1) to test the validity of this record and (2) to test models including an occurrence as close as possible to the Venezuela border in order to “force” the ENMs to predict suitable areas in that country.

## Taxon treatments

### Lesbia nuna

#### Materials

- **Type status:** Other material. **Occurrence:** associatedReferences: Agudelo-Álvarez et al. (2010); occurrenceID: AgudeloAlvarezETAL201001; **Taxon:** scientificName: Lesbia nuna; **Location:** country: Colombia; county: Bogota DC; locality: Campus Pontífica Universidad Javeriana; decimalLatitude: 4.629340; decimalLongitude: -74.064932
- **Type status:** Other material. **Occurrence:** associatedReferences: Peraza (2011); occurrenceID: Peraza201101; **Taxon:** scientificName: Lesbia nuna; **Location:** country: Colombia; county: Bogota DC; locality: Cerros Orientales; decimalLatitude: 4.693056; decimalLongitude: -74.024167
- **Type status:** Other material. **Occurrence:** associatedReferences: Peraza (2011); occurrenceID: Peraza201102; **Taxon:** scientificName: Lesbia nuna; **Location:** country: Colombia; county: Bogota DC; locality: Cerros Orientales; decimalLatitude: 4.689722; decimalLongitude: -74.018056
- **Type status:** Other material. **Occurrence:** associatedReferences: Peraza (2011); occurrenceID: Peraza201103; **Taxon:** scientificName: Lesbia nuna; **Location:** country: Colombia; county: Bogota DC; locality: Cerros Orientales; decimalLatitude: 4.698889; decimalLongitude: -74.013889
- **Type status:** Other material. **Occurrence:** associatedReferences: Peraza (2011); occurrenceID: Peraza201104; **Taxon:** scientificName: Lesbia nuna; **Location:** country: Colombia; county: Bogota DC; locality: Cerros Orientales; decimalLatitude: 4.712778; decimalLongitude: -74.010278
- **Type status:** Other material. **Occurrence:** catalogNumber: MHNCSJ-908; occurrenceID: MHNCSJ908; **Taxon:** scientificName: Lesbia nuna; **Location:** country: Colombia; county: Bogota DC; locality: Chapinero; decimalLatitude: 4.651711; decimalLongitude: -74.061979; **Record Level:** collectionID: Museo de Historia Natural Colegio San José; collectionCode: MHNCSJ
- **Type status:** Other material. **Occurrence:** catalogNumber: MHNCSJ-0934A; occurrenceID: MHNCSJ0934A; **Taxon:** scientificName: Lesbia nuna; **Location:** country: Colombia; county: Bogota DC; locality: Chapinero; decimalLatitude: 4.651711; decimalLongitude: -74.061979; **Record Level:** collectionID: Museo de Historia Natural Colegio San José; collectionCode: MHNCSJ
- **Type status:** Other material. **Occurrence:** catalogNumber: MHNCSJ-0935A; occurrenceID: MHNCSJ0935A; **Taxon:** scientificName: Lesbia nuna; **Location:** country: Colombia; county: Bogota DC; locality: Chapinero; decimalLatitude: 4.651711; decimalLongitude: -74.061979; **Record Level:** collectionID: Museo de Historia Natural Colegio San José; collectionCode: MHNCSJ
- **Type status:** Other material. **Occurrence:** catalogNumber: MHNCSJ-0936A; occurrenceID: MHNCSJ0936A; **Taxon:** scientificName: Lesbia nuna; **Location:** country: Colombia; county: Bogota DC; locality: Chapinero; decimalLatitude: 4.651711; decimalLongitude: -74.061979; **Record Level:** collectionID: Museo de Historia Natural Colegio San José; collectionCode: MHNCSJ
- **Type status:** Other material. **Occurrence:** catalogNumber: MHNCSJ-0936B; occurrenceID: MHNCSJ0936B; **Taxon:** scientificName: Lesbia nuna; **Location:** country: Colombia; county: Bogota DC; locality: Chapinero; decimalLatitude: 4.651711; decimalLongitude: -74.061979; **Record Level:** collectionID: Museo de Historia Natural Colegio San José; collectionCode: MHNCSJ
- **Type status:** Other material. **Occurrence:** catalogNumber: MHNCSJ-0937A; occurrenceID: MHNCSJ0937A; **Taxon:** scientificName: Lesbia nuna; **Location:** country: Colombia; county: Bogota DC; locality: Chapinero; decimalLatitude: 4.651711; decimalLongitude: -74.061979; **Record Level:** collectionID: Museo de Historia Natural Colegio San José; collectionCode: MHNCSJ
- **Type status:** Other material. **Occurrence:** catalogNumber: MLS-2405; occurrenceID: MLS2405; **Taxon:** scientificName: Lesbia nuna; **Location:** country: Colombia; county: Bogota DC; locality: Chapinero; decimalLatitude: 4.653764; decimalLongitude: -74.064726; **Record Level:** collectionID: Universidad de la Salle; collectionCode: MLS
- **Type status:** Other material. **Occurrence:** catalogNumber: ICN-7818; occurrenceID: ICN7818; **Taxon:** scientificName: Lesbia nuna; **Location:** country: Colombia; county: Bogota DC; locality: El Chicó, Bosque; decimalLatitude: 4.675878; decimalLongitude: -74.054810; **Record Level:** collectionID: Instituto de Ciencias Naturales; collectionCode: ICN
- **Type status:** Other material. **Occurrence:** catalogNumber: ICN-7823; occurrenceID: ICN7823; **Taxon:** scientificName: Lesbia nuna; **Location:** country: Colombia; county: Bogota DC; locality: La Cita; decimalLatitude: 4.750000; decimalLongitude: -74.033300; **Record Level:** collectionID: Instituto de Ciencias Naturales; collectionCode: ICN
- **Type status:** Other material. **Occurrence:** catalogNumber: MLS-2408; occurrenceID: MLS2408; **Taxon:** scientificName: Lesbia nuna; **Location:** country: Colombia; county: Bogota DC; locality: La Floresta, (Usaquén); decimalLatitude: 4.700000; decimalLongitude: -74.033300; **Record Level:** collectionID: Universidad de la Salle; collectionCode: MLS
- **Type status:** Other material. **Occurrence:** catalogNumber: MLS-2408; occurrenceID: MLS2408; **Taxon:** scientificName: Lesbia nuna; **Location:** country: Colombia; county: Bogota DC; locality: La Floresta, (Usaquén); decimalLatitude: 4.700000; decimalLongitude: -74.033300; **Record Level:** collectionID: Universidad de la Salle; collectionCode: MLS
- **Type status:** Other material. **Occurrence:** catalogNumber: MHNCSJ-0840A; occurrenceID: MHNCSJ0840A; **Taxon:** scientificName: Lesbia nuna; **Location:** country: Colombia; county: Bogota DC; locality: San Cristobal Boqueron; decimalLatitude: 4.566600; decimalLongitude: -74.083300; **Record Level:** collectionID: Museo de Historia Natural Colegio San José; collectionCode: MHNCSJ
- **Type status:** Other material. **Occurrence:** catalogNumber: MHNCSJ-839; occurrenceID: MHNCSJ839; **Taxon:** scientificName: Lesbia nuna; **Location:** country: Colombia; county: Bogota DC; locality: San Cristobal Boqueron; decimalLatitude: 4.566600; decimalLongitude: -74.083300; **Record Level:** collectionID: Museo de Historia Natural Colegio San José; collectionCode: MHNCSJ
- **Type status:** Other material. **Occurrence:** catalogNumber: MLS-2404; occurrenceID: MLS2404; **Taxon:** scientificName: Lesbia nuna; **Location:** country: Colombia; county: Bogota DC; locality: Usaquén (and "Usaquen, en la Sabana"); decimalLatitude: 4.700000; decimalLongitude: -74.033300; **Record Level:** collectionID: Universidad de la Salle; collectionCode: MLS
- **Type status:** Other material. **Occurrence:** catalogNumber: MLS-2406; occurrenceID: MLS2406; **Taxon:** scientificName: Lesbia nuna; **Location:** country: Colombia; county: Bogota DC; locality: Usaquén (and "Usaquen, en la Sabana"); decimalLatitude: 4.700000; decimalLongitude: -74.033300; **Record Level:** collectionID: Universidad de la Salle; collectionCode: MLS
- **Type status:** Other material. **Occurrence:** catalogNumber: UNIANDES-W/No.; occurrenceID: UNIANDESNONUMBER; **Taxon:** scientificName: Lesbia nuna; **Location:** country: Colombia; county: Bogota DC; locality: Usaquén (and "Usaquen, en la Sabana"); decimalLatitude: 4.700000; decimalLongitude: -74.033300; **Record Level:** collectionID: Universidad de los Andes; collectionCode: UNIANDES
- **Type status:** Other material. **Occurrence:** catalogNumber: IAvH-6138; occurrenceID: IAvH6138; **Taxon:** scientificName: Lesbia nuna; **Location:** country: Colombia; county: Bogota DC; locality: Vivero Venado de Oro; decimalLatitude: 4.616990; decimalLongitude: -74.064060; **Record Level:** collectionID: Instituto Alexander von Humboldt; collectionCode: IAvH
- **Type status:** Other material. **Occurrence:** associatedReferences: Andrade & Benitez -Castańeda (2012); occurrenceID: AndradeANDBenitezCastańeda201201; **Taxon:** scientificName: Lesbia nuna; **Location:** country: Colombia; county: Bogota DC; locality: [Suba] Humedal Córdoba; decimalLatitude: 4.717409; decimalLongitude: -74.071884
- **Type status:** Other material. **Occurrence:** associatedReferences: Andrade & Benitez -Castańeda (2012); occurrenceID: AndradeANDBenitezCastańeda201202; **Taxon:** scientificName: Lesbia nuna; **Location:** country: Colombia; county: Bogota DC; locality: [Suba] Humedal La Conejera; decimalLatitude: 4.781048; decimalLongitude: -74.065361
- **Type status:** Other material. **Occurrence:** associatedReferences: Andrade & Benitez -Castańeda (2012); occurrenceID: AndradeANDBenitezCastańeda201203; **Taxon:** scientificName: Lesbia nuna; **Location:** country: Colombia; county: Bogota DC; locality: [Torca] Humedal de Torca; decimalLatitude: 4.809401; decimalLongitude: -74.040813
- **Type status:** Other material. **Occurrence:** catalogNumber: ICN-7817; occurrenceID: ICN7817; **Taxon:** scientificName: Lesbia nuna; **Location:** country: Colombia; county: Bogota DC; locality: El Prado, Sabana de Bogotá, 5 k al norte de Bogotá, Lag. El Prado; decimalLatitude: 4.716600; decimalLongitude: -74.066600; **Record Level:** collectionID: Instituto de Ciencias Naturales; collectionCode: ICN
- **Type status:** Other material. **Occurrence:** catalogNumber: MVZ-120505; occurrenceID: MVZ120505; **Taxon:** scientificName: Lesbia nuna; **Location:** country: Colombia; stateProvince: Boyaca; municipality: Chiquinquirá; locality: Chiquinquirá, 10 KM ESE; decimalLatitude: 5.557898; decimalLongitude: -73.793158; **Record Level:** collectionID: Museum of Vertebrate Zoology University of California Berkeley; collectionCode: MVZ
- **Type status:** Other material. **Occurrence:** associatedMedia: Internet Bird Collection (http://ibc.lynxeds.com/photo/green-tailed-trainbearer-lesbia-nuna/male-feeding-blackberry-flower); occurrenceID: InternetBirdCollectionLesbiaNunaMale01; **Taxon:** scientificName: Lesbia nuna; **Location:** country: Colombia; stateProvince: Boyaca; municipality: Jenesano; locality: Tibaná; decimalLatitude: 5.316600; decimalLongitude: -73.383300
- **Type status:** Other material. **Occurrence:** catalogNumber: Xeno-Canto-79764; occurrenceID: XenoCanto79764; **Taxon:** scientificName: Lesbia nuna; **Location:** country: Colombia; stateProvince: Boyaca; municipality: Miraflores; locality: Miraflores; decimalLatitude: 5.197629; decimalLongitude: -73.198574
- **Type status:** Other material. **Occurrence:** catalogNumber: MHN-UCC-W/No.; occurrenceID: MHNUCCNONUMBER; **Taxon:** scientificName: Lesbia nuna; **Location:** country: Colombia; stateProvince: Boyaca; municipality: Sogamoso; locality: Sogamoso; decimalLatitude: 5.716600; decimalLongitude: -72.933300; **Record Level:** collectionID: Universidad del Cauca; collectionCode: MHN-UCC
- **Type status:** Other material. **Occurrence:** associatedReferences: Olivares (1966); occurrenceID: Olivares196601; **Taxon:** scientificName: Lesbia nuna; **Location:** country: Colombia; stateProvince: Boyaca; municipality: Tunja; locality: Motavita; decimalLatitude: 5.583300; decimalLongitude: -73.383300
- **Type status:** Other material. **Occurrence:** catalogNumber: USNM - 446180; occurrenceID: USNM446180; **Taxon:** scientificName: Lesbia nuna; **Location:** country: Colombia; stateProvince: Boyaca; municipality: Popayán; locality: Cerro Aguablanca; decimalLatitude: 2.246129; decimalLongitude: -76.396385; **Record Level:** collectionID: National Museum of Natural History; collectionCode: USNM
- **Type status:** Other material. **Occurrence:** catalogNumber: NHM - 1933.11.14.3; occurrenceID: NHM1933.11.14.3; **Taxon:** scientificName: Lesbia nuna; **Location:** country: Colombia; stateProvince: Boyaca; municipality: Popayán; locality: Popayán; decimalLatitude: 2.450000; decimalLongitude: -76.600000; **Record Level:** collectionID: Natural History Museum London; collectionCode: NHM
- **Type status:** Other material. **Occurrence:** catalogNumber: WFVZ - 9344; occurrenceID: WFVZ 9344; **Taxon:** scientificName: Lesbia nuna; **Location:** country: Colombia; stateProvince: Boyaca; municipality: Popayán; locality: Popayán; decimalLatitude: 2.450000; decimalLongitude: -76.600000; **Record Level:** collectionID: Western Foundation of Vertebrate Zoology; collectionCode: WFVZ
- **Type status:** Other material. **Occurrence:** associatedReferences: Olivares (1966); occurrenceID: Olivares196602; **Taxon:** scientificName: Lesbia nuna; **Location:** country: Colombia; stateProvince: Boyaca; municipality: Popayán; locality: Puracé; decimalLatitude: 2.383300; decimalLongitude: -76.450000
- **Type status:** Other material. **Occurrence:** catalogNumber: USNM-446179; occurrenceID: USNM446179; **Taxon:** scientificName: Lesbia nuna; **Location:** country: Colombia; stateProvince: Boyaca; municipality: Popayán; locality: Puracé; decimalLatitude: 2.383300; decimalLongitude: -76.450000; **Record Level:** collectionID: National Museum of Natural History; collectionCode: USNM
- **Type status:** Other material. **Occurrence:** catalogNumber: USNM-446181; occurrenceID: USNM446181; **Taxon:** scientificName: Lesbia nuna; **Location:** country: Colombia; stateProvince: Boyaca; municipality: Popayán; locality: Puracé; decimalLatitude: 2.383300; decimalLongitude: -76.450000; **Record Level:** collectionID: National Museum of Natural History; collectionCode: USNM
- **Type status:** Other material. **Occurrence:** catalogNumber: USNM-446182; occurrenceID: USNM446182; **Taxon:** scientificName: Lesbia nuna; **Location:** country: Colombia; stateProvince: Boyaca; municipality: Popayán; locality: Puracé; decimalLatitude: 2.383300; decimalLongitude: -76.450000; **Record Level:** collectionID: National Museum of Natural History; collectionCode: USNM
- **Type status:** Other material. **Occurrence:** catalogNumber: USNM-446183; occurrenceID: USNM446183; **Taxon:** scientificName: Lesbia nuna; **Location:** country: Colombia; stateProvince: Boyaca; municipality: Popayán; locality: Puracé; decimalLatitude: 2.383300; decimalLongitude: -76.450000; **Record Level:** collectionID: National Museum of Natural History; collectionCode: USNM
- **Type status:** Other material. **Occurrence:** catalogNumber: USNM-446184; occurrenceID: USNM446184; **Taxon:** scientificName: Lesbia nuna; **Location:** country: Colombia; stateProvince: Boyaca; municipality: Popayán; locality: Puracé; decimalLatitude: 2.383300; decimalLongitude: -76.450000; **Record Level:** collectionID: National Museum of Natural History; collectionCode: USNM
- **Type status:** Other material. **Occurrence:** catalogNumber: USNM-446185; occurrenceID: USNM446185; **Taxon:** scientificName: Lesbia nuna; **Location:** country: Colombia; stateProvince: Boyaca; municipality: Popayán; locality: Puracé; decimalLatitude: 2.383300; decimalLongitude: -76.450000; **Record Level:** collectionID: National Museum of Natural History; collectionCode: USNM
- **Type status:** Other material. **Occurrence:** catalogNumber: RMNH-8172; occurrenceID: RMNH8172; **Taxon:** scientificName: Lesbia nuna; **Location:** country: Colombia; stateProvince: Boyaca; municipality: Popayán; locality: Puracé; decimalLatitude: 2.383300; decimalLongitude: -76.450000; **Record Level:** collectionID: Nationaal Natuurhistorisch Museum; collectionCode: RMNH
- **Type status:** Other material. **Occurrence:** catalogNumber: YPM-026942; occurrenceID: YPM026942; **Taxon:** scientificName: Lesbia nuna; **Location:** country: Colombia; stateProvince: Boyaca; municipality: Popayán; locality: Puracé; decimalLatitude: 2.383300; decimalLongitude: -76.450000; **Record Level:** collectionID: Yale Peabody Museum; collectionCode: YPM
- **Type status:** Other material. **Occurrence:** catalogNumber: YPM-026943; occurrenceID: YPM026943; **Taxon:** scientificName: Lesbia nuna; **Location:** country: Colombia; stateProvince: Boyaca; municipality: Popayán; locality: Puracé; decimalLatitude: 2.383300; decimalLongitude: -76.450000; **Record Level:** collectionID: Yale Peabody Museum; collectionCode: YPM
- **Type status:** Other material. **Occurrence:** occurrenceRemarks: Excluded from ENMs; associatedReferences: Olivares (1966); occurrenceID: Olivares196603; **Taxon:** scientificName: Lesbia nuna; **Location:** country: Colombia; stateProvince: Boyaca; municipality: Popayán; locality: Coconuco; decimalLatitude: 2.350000; decimalLongitude: -76.500000
- **Type status:** Other material. **Occurrence:** catalogNumber: INCIVA-1020; occurrenceRemarks: Excluded from ENMs; occurrenceID: INCIVA1020; **Taxon:** scientificName: Lesbia nuna; **Location:** country: Colombia; stateProvince: Boyaca; municipality: Popayán; locality: Coconuco; decimalLatitude: 2.350000; decimalLongitude: -76.500000; **Record Level:** collectionID: Instituto Vallecaucano de Investigaciones; collectionCode: INCIVA
- **Type status:** Other material. **Occurrence:** catalogNumber: INCIVA-1021; occurrenceRemarks: Excluded from ENMs; occurrenceID: INCIVA1021; **Taxon:** scientificName: Lesbia nuna; **Location:** country: Colombia; stateProvince: Boyaca; municipality: Popayán; locality: Coconuco; decimalLatitude: 2.350000; decimalLongitude: -76.500000; **Record Level:** collectionID: Instituto Vallecaucano de Investigaciones; collectionCode: INCIVA
- **Type status:** Other material. **Occurrence:** catalogNumber: INCIVA-1022; occurrenceRemarks: Excluded from ENMs; occurrenceID: INCIVA1022; **Taxon:** scientificName: Lesbia nuna; **Location:** country: Colombia; stateProvince: Boyaca; municipality: Popayán; locality: Coconuco; decimalLatitude: 2.350000; decimalLongitude: -76.500000; **Record Level:** collectionID: Instituto Vallecaucano de Investigaciones; collectionCode: INCIVA
- **Type status:** Other material. **Occurrence:** catalogNumber: INCIVA-1023; occurrenceRemarks: Excluded from ENMs; occurrenceID: INCIVA1023; **Taxon:** scientificName: Lesbia nuna; **Location:** country: Colombia; stateProvince: Boyaca; municipality: Popayán; locality: Coconuco; decimalLatitude: 2.350000; decimalLongitude: -76.500000; **Record Level:** collectionID: Instituto Vallecaucano de Investigaciones; collectionCode: INCIVA
- **Type status:** Other material. **Occurrence:** catalogNumber: RMNH-8172; occurrenceRemarks: Excluded from ENMs; occurrenceID: RMNH8172; **Taxon:** scientificName: Lesbia nuna; **Location:** country: Colombia; stateProvince: Boyaca; municipality: Popayán; locality: Coconuco; decimalLatitude: 2.350000; decimalLongitude: -76.500000; **Record Level:** collectionID: Naturalis Biodiversity Center; collectionCode: RMNH
- **Type status:** Other material. **Occurrence:** catalogNumber: MHN-UCC-3623; occurrenceRemarks: Excluded from ENMs; occurrenceID: MHNUCC3623; **Taxon:** scientificName: Lesbia nuna; **Location:** country: Colombia; stateProvince: Boyaca; municipality: Popayán; locality: Coconuco; decimalLatitude: 2.350000; decimalLongitude: -76.500000; **Record Level:** collectionID: Universidad del Cauca; collectionCode: MHN-UCC
- **Type status:** Other material. **Occurrence:** catalogNumber: MHN-UCC-W/No.; occurrenceRemarks: Excluded from ENMs; occurrenceID: MHNUCCNONUMBER; **Taxon:** scientificName: Lesbia nuna; **Location:** country: Colombia; stateProvince: Boyaca; municipality: Popayán; locality: Coconuco; decimalLatitude: 2.350000; decimalLongitude: -76.500000; **Record Level:** collectionID: Universidad del Cauca; collectionCode: MHN-UCC
- **Type status:** Other material. **Occurrence:** catalogNumber: LACM-36250; occurrenceRemarks: Excluded from ENMs; occurrenceID: LACM36250; **Taxon:** scientificName: Lesbia nuna; **Location:** country: Colombia; stateProvince: Boyaca; municipality: Sotará; locality: El Crucero; decimalLatitude: 2.383300; decimalLongitude: -76.650000; **Record Level:** collectionID: Natural History Museum of Los Angeles County; collectionCode: LACM
- **Type status:** Other material. **Occurrence:** catalogNumber: ROM-101252; occurrenceID: ROM101252; **Taxon:** scientificName: Lesbia nuna; **Location:** country: Colombia; stateProvince: Boyaca; municipality: Timbío; locality: Chiribio; decimalLatitude: 2.350000; decimalLongitude: -76.716600; **Record Level:** collectionID: Royal Ontario Museum; collectionCode: ROM
- **Type status:** Other material. **Occurrence:** catalogNumber: ROM-114827; occurrenceID: ROM114827; **Taxon:** scientificName: Lesbia nuna; **Location:** country: Colombia; stateProvince: Boyaca; municipality: Timbío; locality: Chiribio; decimalLatitude: 2.350000; decimalLongitude: -76.716600; **Record Level:** collectionID: Royal Ontario Museum; collectionCode: ROM
- **Type status:** Other material. **Occurrence:** catalogNumber: MHN-UCC-4341; occurrenceRemarks: Excluded from ENMs; occurrenceID: MHNUCC4341; **Taxon:** scientificName: Lesbia nuna; **Location:** country: Colombia; stateProvince: Boyaca; municipality: Totoró [Inzá]; locality: Loma Alta, [río] Malvasá, Cauca; decimalLatitude: 2.557500; decimalLongitude: -76.070200; **Record Level:** collectionID: Universidad del Cauca; collectionCode: MHN-UCC
- **Type status:** Other material. **Occurrence:** catalogNumber: ICN-24092; occurrenceID: ICN24092; **Taxon:** scientificName: Lesbia nuna; **Location:** country: Colombia; stateProvince: Cundinamarca; municipality: Chía; locality: Guaymaral; decimalLatitude: 4.835893; decimalLongitude: -74.070514; **Record Level:** collectionID: Instituto de Ciencias Naturales; collectionCode: ICN
- **Type status:** Other material. **Occurrence:** catalogNumber: ICN-20437; occurrenceID: ICN20437; **Taxon:** scientificName: Lesbia nuna; **Location:** country: Colombia; stateProvince: Cundinamarca; municipality: Chía; locality: Estación La Caro; decimalLatitude: 4.868584; decimalLongitude: -74.033392; **Record Level:** collectionID: Instituto de Ciencias Naturales; collectionCode: ICN
- **Type status:** Other material. **Occurrence:** associatedReferences: Echeverry-Galvis & Morales-Rozo (2007); occurrenceID: EcheverryGalvisANDMoralesRozo200701; **Taxon:** scientificName: Lesbia nuna; **Location:** country: Colombia; stateProvince: Cundinamarca; municipality: Chía; locality: Vereda Cerca de Piedra; decimalLatitude: 4.850000; decimalLongitude: -74.050000
- **Type status:** Other material. **Occurrence:** catalogNumber: ANS-149171; occurrenceID: ANS149171; **Taxon:** scientificName: Lesbia nuna; **Location:** country: Colombia; stateProvince: Cundinamarca; municipality: Choachí; locality: Choachí; decimalLatitude: 4.533300; decimalLongitude: -73.933300; **Record Level:** collectionID: Academy of Natural Sciences Philadelphia; collectionCode: ANS
- **Type status:** Other material. **Occurrence:** catalogNumber: ANS-149172; occurrenceID: ANS149172; **Taxon:** scientificName: Lesbia nuna; **Location:** country: Colombia; stateProvince: Cundinamarca; municipality: Choachí; locality: Choachí; decimalLatitude: 4.533300; decimalLongitude: -73.933300; **Record Level:** collectionID: Academy of Natural Sciences Philadelphia; collectionCode: ANS
- **Type status:** Other material. **Occurrence:** catalogNumber: ANS-149173; occurrenceID: ANS149173; **Taxon:** scientificName: Lesbia nuna; **Location:** country: Colombia; stateProvince: Cundinamarca; municipality: Choachí; locality: Choachí; decimalLatitude: 4.533300; decimalLongitude: -73.933300; **Record Level:** collectionID: Academy of Natural Sciences Philadelphia; collectionCode: ANS
- **Type status:** Other material. **Occurrence:** catalogNumber: ICN-7819; occurrenceID: ICN7819; **Taxon:** scientificName: Lesbia nuna; **Location:** country: Colombia; stateProvince: Cundinamarca; municipality: Chocontá; locality: Represa del Sisga; decimalLatitude: 5.083300; decimalLongitude: -73.716600; **Record Level:** collectionID: Instituto de Ciencias Naturales; collectionCode: ICN
- **Type status:** Other material. **Occurrence:** catalogNumber: ICN-7820; occurrenceID: ICN7820; **Taxon:** scientificName: Lesbia nuna; **Location:** country: Colombia; stateProvince: Cundinamarca; municipality: Chocontá; locality: Represa del Sisga; decimalLatitude: 5.083300; decimalLongitude: -73.716600; **Record Level:** collectionID: Instituto de Ciencias Naturales; collectionCode: ICN
- **Type status:** Other material. **Occurrence:** catalogNumber: ICN-7824; occurrenceID: ICN7824; **Taxon:** scientificName: Lesbia nuna; **Location:** country: Colombia; stateProvince: Cundinamarca; municipality: Chocontá; locality: Represa del Sisga; decimalLatitude: 5.083300; decimalLongitude: -73.716600; **Record Level:** collectionID: Instituto de Ciencias Naturales; collectionCode: ICN
- **Type status:** Other material. **Occurrence:** associatedReferences: Andrade & Benitez -Castańeda (2012); occurrenceID: AndradeANDBenitezCastańeda201204; **Taxon:** scientificName: Lesbia nuna; **Location:** country: Colombia; stateProvince: Cundinamarca; municipality: Cota; locality: Parque La Florida; decimalLatitude: 4.736399; decimalLongitude: -74.146557
- **Type status:** Other material. **Occurrence:** catalogNumber: ANS-167969; occurrenceID: ANS167969; **Taxon:** scientificName: Lesbia nuna; **Location:** country: Colombia; stateProvince: Cundinamarca; municipality: Gachancipá; locality: Gachancipá; decimalLatitude: 5.000000; decimalLongitude: -73.883300; **Record Level:** collectionID: Academy of Natural Sciences Philadelphia; collectionCode: ANS
- **Type status:** Other material. **Occurrence:** catalogNumber: ANS-167979; occurrenceID: ANS167979; **Taxon:** scientificName: Lesbia nuna; **Location:** country: Colombia; stateProvince: Cundinamarca; municipality: Gachancipá; locality: Gachancipá; decimalLatitude: 5.000000; decimalLongitude: -73.883300; **Record Level:** collectionID: Academy of Natural Sciences Philadelphia; collectionCode: ANS
- **Type status:** Other material. **Occurrence:** catalogNumber: ICN-7821; occurrenceID: ICN7821; **Taxon:** scientificName: Lesbia nuna; **Location:** country: Colombia; stateProvince: Cundinamarca; municipality: Gachancipá; locality: Gachancipá; decimalLatitude: 5.000000; decimalLongitude: -73.883300; **Record Level:** collectionID: Instituto de Ciencias Naturales; collectionCode: ICN
- **Type status:** Other material. **Occurrence:** catalogNumber: ICN-7822; occurrenceID: ICN7822; **Taxon:** scientificName: Lesbia nuna; **Location:** country: Colombia; stateProvince: Cundinamarca; municipality: Gachancipá; locality: Gachancipá; decimalLatitude: 5.000000; decimalLongitude: -73.883300; **Record Level:** collectionID: Instituto de Ciencias Naturales; collectionCode: ICN
- **Type status:** Other material. **Occurrence:** catalogNumber: MCZ-343415; occurrenceID: MCZ343415; **Taxon:** scientificName: Lesbia nuna; **Location:** country: Colombia; stateProvince: Cundinamarca; municipality: Gachancipá; locality: Gachancipá; decimalLatitude: 5.000000; decimalLongitude: -73.883300; **Record Level:** collectionID: Museum of Comparative Zoology Harvard University; collectionCode: MCZ
- **Type status:** Other material. **Occurrence:** catalogNumber: ICN-20152; occurrenceID: ICN20152; **Taxon:** scientificName: Lesbia nuna; **Location:** country: Colombia; stateProvince: Cundinamarca; municipality: Guasca; locality: Carretera a Guasca; decimalLatitude: 4.879573; decimalLongitude: -73.883160; **Record Level:** collectionID: Instituto de Ciencias Naturales; collectionCode: ICN
- **Type status:** Other material. **Occurrence:** catalogNumber: MLS-2402; occurrenceID: MLS2402; **Taxon:** scientificName: Lesbia nuna; **Location:** country: Colombia; stateProvince: Cundinamarca; municipality: Guasca; locality: Sopó (includes "Valle de Sopó"); decimalLatitude: 4.916600; decimalLongitude: -73.916600; **Record Level:** collectionID: Universidad de la Salle; collectionCode: MLS
- **Type status:** Other material. **Occurrence:** catalogNumber: MLS-2403; occurrenceID: MLS2403; **Taxon:** scientificName: Lesbia nuna; **Location:** country: Colombia; stateProvince: Cundinamarca; municipality: Guasca; locality: Sopó (includes "Valle de Sopó"); decimalLatitude: 4.916600; decimalLongitude: -73.916600; **Record Level:** collectionID: Universidad de la Salle; collectionCode: MLS
- **Type status:** Other material. **Occurrence:** catalogNumber: ICN-7813; occurrenceID: ICN7813; **Taxon:** scientificName: Lesbia nuna; **Location:** country: Colombia; stateProvince: Cundinamarca; municipality: Guasca; locality: Sopó (includes "Valle de Sopó"); decimalLatitude: 4.916600; decimalLongitude: -73.916600; **Record Level:** collectionID: Instituto de Ciencias Naturales; collectionCode: ICN
- **Type status:** Other material. **Occurrence:** catalogNumber: ICN-7814; occurrenceID: ICN7814; **Taxon:** scientificName: Lesbia nuna; **Location:** country: Colombia; stateProvince: Cundinamarca; municipality: Guasca; locality: Sopó (includes "Valle de Sopó"); decimalLatitude: 4.916600; decimalLongitude: -73.916600; **Record Level:** collectionID: Instituto de Ciencias Naturales; collectionCode: ICN
- **Type status:** Other material. **Occurrence:** catalogNumber: ICN-33661; occurrenceID: ICN33661; **Taxon:** scientificName: Lesbia nuna; **Location:** country: Colombia; stateProvince: Cundinamarca; municipality: Guasca; locality: Vda. La Floresta, Fca. La Plata, margen izq. Río Chipatá; decimalLatitude: 4.824200; decimalLongitude: -73.838500; **Record Level:** collectionID: Instituto de Ciencias Naturales; collectionCode: ICN
- **Type status:** Other material. **Occurrence:** associatedReferences: Amaya-Villarreal & Renjifo (2010); occurrenceID: AmayaVillarrealANDRenjifo201001; **Taxon:** scientificName: Lesbia nuna; **Location:** country: Colombia; stateProvince: Cundinamarca; municipality: Guasca; locality: Reserva Biológica el Encenillo; decimalLatitude: 4.841687; decimalLongitude: -73.899978
- **Type status:** Other material. **Occurrence:** catalogNumber: ICN-15590; occurrenceID: ICN15590; **Taxon:** scientificName: Lesbia nuna; **Location:** country: Colombia; stateProvince: Cundinamarca; municipality: Guatavita; locality: Guatavita; decimalLatitude: 4.916600; decimalLongitude: -73.850000; **Record Level:** collectionID: Instituto de Ciencias Naturales; collectionCode: ICN
- **Type status:** Other material. **Occurrence:** associatedReferences: Weller & Schuchmann (2004); occurrenceID: WellerANDSchuchmann200401; **Taxon:** scientificName: Lesbia nuna; **Location:** country: Colombia; stateProvince: Cundinamarca; municipality: Pacho; locality: Mountains of Pacho; decimalLatitude: 5.100000; decimalLongitude: -74.166670
- **Type status:** Other material. **Occurrence:** catalogNumber: ROM-105389; occurrenceID: ROM105389; **Taxon:** scientificName: Lesbia nuna; **Location:** country: Colombia; stateProvince: Cundinamarca; municipality: Sibaté; locality: 15 km N Sibate, San Benito Seminario Menor de Bogotá; decimalLatitude: 4.508532; decimalLongitude: -74.245784; **Record Level:** collectionID: Royal Ontario Museum; collectionCode: ROM
- **Type status:** Other material. **Occurrence:** catalogNumber: AMNH-121656; occurrenceID: AMNH121656; **Taxon:** scientificName: Lesbia nuna; **Location:** country: Colombia; stateProvince: Cundinamarca; municipality: Sibaté; locality: Sibaté (includes "Sibate above Bogotá"); decimalLatitude: 4.491300; decimalLongitude: -74.260500; **Record Level:** collectionID: American Museum of Natural History; collectionCode: AMNH
- **Type status:** Other material. **Occurrence:** catalogNumber: MLS-2400; occurrenceID: MLS2400; **Taxon:** scientificName: Lesbia nuna; **Location:** country: Colombia; stateProvince: Cundinamarca; municipality: Sibaté; locality: Sibaté (includes "Sibate above Bogotá"); decimalLatitude: 4.491300; decimalLongitude: -74.260500; **Record Level:** collectionID: Universidad de la Salle; collectionCode: MLS
- **Type status:** Other material. **Occurrence:** catalogNumber: MLS-2401; occurrenceID: MLS2401; **Taxon:** scientificName: Lesbia nuna; **Location:** country: Colombia; stateProvince: Cundinamarca; municipality: Sibaté; locality: Sibaté (includes "Sibate above Bogotá"); decimalLatitude: 4.491300; decimalLongitude: -74.260500; **Record Level:** collectionID: Universidad de la Salle; collectionCode: MLS
- **Type status:** Other material. **Occurrence:** catalogNumber: AMNH-126477; occurrenceID: AMNH126477; **Taxon:** scientificName: Lesbia nuna; **Location:** country: Colombia; stateProvince: Cundinamarca; municipality: Subachoque; locality: La Mar; decimalLatitude: 4.933300; decimalLongitude: -74.183300; **Record Level:** collectionID: American Museum of Natural History; collectionCode: AMNH
- **Type status:** Other material. **Occurrence:** catalogNumber: MLS-2397; occurrenceID: MLS2397; **Taxon:** scientificName: Lesbia nuna; **Location:** country: Colombia; stateProvince: Cundinamarca; municipality: Subachoque; locality: Subachoque; decimalLatitude: 4.933300; decimalLongitude: -74.183300; **Record Level:** collectionID: Universidad de la Salle; collectionCode: MLS
- **Type status:** Other material. **Occurrence:** catalogNumber: MLS-2398; occurrenceID: MLS2398; **Taxon:** scientificName: Lesbia nuna; **Location:** country: Colombia; stateProvince: Cundinamarca; municipality: Subachoque; locality: Subachoque; decimalLatitude: 4.933300; decimalLongitude: -74.183300; **Record Level:** collectionID: Universidad de la Salle; collectionCode: MLS
- **Type status:** Other material. **Occurrence:** catalogNumber: MLS-2399; occurrenceID: MLS2399; **Taxon:** scientificName: Lesbia nuna; **Location:** country: Colombia; stateProvince: Cundinamarca; municipality: Subachoque; locality: Subachoque; decimalLatitude: 4.933300; decimalLongitude: -74.183300; **Record Level:** collectionID: Universidad de la Salle; collectionCode: MLS
- **Type status:** Other material. **Occurrence:** catalogNumber: FMNH-220400; occurrenceID: FMNH220400; **Taxon:** scientificName: Lesbia nuna; **Location:** country: Colombia; stateProvince: Cundinamarca; municipality: Susa; locality: Laguna de Fuquene; decimalLatitude: 5.466600; decimalLongitude: -73.750000; **Record Level:** collectionID: Field Museum of Natural History; collectionCode: FMNH
- **Type status:** Other material. **Occurrence:** catalogNumber: FMNH-220401; occurrenceID: FMNH220401; **Taxon:** scientificName: Lesbia nuna; **Location:** country: Colombia; stateProvince: Cundinamarca; municipality: Susa; locality: Laguna de Fuquene; decimalLatitude: 5.466600; decimalLongitude: -73.750000; **Record Level:** collectionID: Field Museum of Natural History; collectionCode: FMNH
- **Type status:** Other material. **Occurrence:** catalogNumber: FMNH-220403; occurrenceID: FMNH220403; **Taxon:** scientificName: Lesbia nuna; **Location:** country: Colombia; stateProvince: Cundinamarca; municipality: Susa; locality: Laguna de Fuquene; decimalLatitude: 5.466600; decimalLongitude: -73.750000; **Record Level:** collectionID: Field Museum of Natural History; collectionCode: FMNH
- **Type status:** Other material. **Occurrence:** catalogNumber: FMNH-220404; occurrenceID: FMNH220404; **Taxon:** scientificName: Lesbia nuna; **Location:** country: Colombia; stateProvince: Cundinamarca; municipality: Susa; locality: Laguna de Fuquene; decimalLatitude: 5.466600; decimalLongitude: -73.750000; **Record Level:** collectionID: Field Museum of Natural History; collectionCode: FMNH
- **Type status:** Other material. **Occurrence:** catalogNumber: FMNH-220405; occurrenceID: FMNH220405; **Taxon:** scientificName: Lesbia nuna; **Location:** country: Colombia; stateProvince: Cundinamarca; municipality: Susa; locality: Laguna de Fuquene; decimalLatitude: 5.466600; decimalLongitude: -73.750000; **Record Level:** collectionID: Field Museum of Natural History; collectionCode: FMNH
- **Type status:** Other material. **Occurrence:** catalogNumber: FMNH-220407; occurrenceID: FMNH220407; **Taxon:** scientificName: Lesbia nuna; **Location:** country: Colombia; stateProvince: Cundinamarca; municipality: Susa; locality: Laguna de Fuquene; decimalLatitude: 5.466600; decimalLongitude: -73.750000; **Record Level:** collectionID: Field Museum of Natural History; collectionCode: FMNH
- **Type status:** Other material. **Occurrence:** catalogNumber: ICN-13406; occurrenceID: ICN13406; **Taxon:** scientificName: Lesbia nuna; **Location:** country: Colombia; stateProvince: Cundinamarca; municipality: Tabio; locality: Tabío, a orillas del Río Frío; decimalLatitude: 4.916600; decimalLongitude: -74.100000; **Record Level:** collectionID: Instituto de Ciencias Naturales; collectionCode: ICN
- **Type status:** Other material. **Occurrence:** catalogNumber: ICN-17169; occurrenceID: ICN17169; **Taxon:** scientificName: Lesbia nuna; **Location:** country: Colombia; stateProvince: Cundinamarca; municipality: Une; locality: Vereda Quimasita [Timasita]; decimalLatitude: 4.425580; decimalLongitude: -74.022600; **Record Level:** collectionID: Instituto de Ciencias Naturales; collectionCode: ICN
- **Type status:** Other material. **Occurrence:** catalogNumber: ICN-17235; occurrenceID: ICN17235; **Taxon:** scientificName: Lesbia nuna; **Location:** country: Colombia; stateProvince: Cundinamarca; municipality: Une; locality: Vereda Quimasita [Timasita]; decimalLatitude: 4.425580; decimalLongitude: -74.022600; **Record Level:** collectionID: Instituto de Ciencias Naturales; collectionCode: ICN
- **Type status:** Other material. **Occurrence:** catalogNumber: ICN-16852; occurrenceID: ICN16852; **Taxon:** scientificName: Lesbia nuna; **Location:** country: Colombia; stateProvince: Cundinamarca; municipality: Une; locality: Vereda Quimasita [Timasita]; decimalLatitude: 4.425580; decimalLongitude: -74.022600; **Record Level:** collectionID: Instituto de Ciencias Naturales; collectionCode: ICN
- **Type status:** Other material. **Occurrence:** catalogNumber: ICN-16854; occurrenceID: ICN16854; **Taxon:** scientificName: Lesbia nuna; **Location:** country: Colombia; stateProvince: Cundinamarca; municipality: Une; locality: Vereda Quimasita [Timasita]; decimalLatitude: 4.425580; decimalLongitude: -74.022600; **Record Level:** collectionID: Instituto de Ciencias Naturales; collectionCode: ICN
- **Type status:** Other material. **Occurrence:** catalogNumber: ICN-17000; occurrenceID: ICN17000; **Taxon:** scientificName: Lesbia nuna; **Location:** country: Colombia; stateProvince: Cundinamarca; municipality: Une; locality: Vereda Quimasita [Timasita]; decimalLatitude: 4.425580; decimalLongitude: -74.022600; **Record Level:** collectionID: Instituto de Ciencias Naturales; collectionCode: ICN
- **Type status:** Other material. **Occurrence:** catalogNumber: DMNH-56774; occurrenceID: DMNH56774; **Taxon:** scientificName: Lesbia nuna; **Location:** country: Colombia; stateProvince: Cundinamarca; municipality: Zipacón; locality: Zipacón; decimalLatitude: 4.766600; decimalLongitude: -74.383300; **Record Level:** collectionID: Delaware Natural History Museum; collectionCode: DMNH
- **Type status:** Other material. **Occurrence:** catalogNumber: DMNH-63282; occurrenceID: DMNH63282; **Taxon:** scientificName: Lesbia nuna; **Location:** country: Colombia; stateProvince: Cundinamarca; municipality: Zipacón; locality: Zipacón; decimalLatitude: 4.766600; decimalLongitude: -74.383300; **Record Level:** collectionID: Delaware Natural History Museum; collectionCode: DMNH
- **Type status:** Other material. **Occurrence:** catalogNumber: ICN-26170; occurrenceID: ICN26170; **Taxon:** scientificName: Lesbia nuna; **Location:** country: Colombia; stateProvince: Huila; municipality: La Plata; locality: Nevado del Huila PNN, Est. Termales; decimalLatitude: 2.945600; decimalLongitude: -76.087900; **Record Level:** collectionID: Instituto de Ciencias Naturales; collectionCode: ICN
- **Type status:** Other material. **Occurrence:** catalogNumber: FMNH-249605; occurrenceID: FMNH249605; **Taxon:** scientificName: Lesbia nuna; **Location:** country: Colombia; stateProvince: Narińo; municipality: Mallama; locality: El Guabo; decimalLatitude: 1.116600; decimalLongitude: -77.816600; **Record Level:** collectionID: Field Museum of Natural History; collectionCode: FMNH
- **Type status:** Other material. **Occurrence:** catalogNumber: LACM-30498; occurrenceID: LACM30498; **Taxon:** scientificName: Lesbia nuna; **Location:** country: Colombia; stateProvince: Narińo; municipality: Mallama; locality: El Guabo; decimalLatitude: 1.116600; decimalLongitude: -77.816600; **Record Level:** collectionID: Natural History Museum of Los Angeles County; collectionCode: LACM
- **Type status:** Other material. **Occurrence:** catalogNumber: LACM-30499; occurrenceID: LACM30499; **Taxon:** scientificName: Lesbia nuna; **Location:** country: Colombia; stateProvince: Narińo; municipality: Mallama; locality: El Guabo; decimalLatitude: 1.116600; decimalLongitude: -77.816600; **Record Level:** collectionID: Natural History Museum of Los Angeles County; collectionCode: LACM
- **Type status:** Other material. **Occurrence:** catalogNumber: WFVZ-12979; occurrenceID: WFVZ12979; **Taxon:** scientificName: Lesbia nuna; **Location:** country: Colombia; stateProvince: Narińo; municipality: Mallama; locality: El Guabo; decimalLatitude: 1.116600; decimalLongitude: -77.816600; **Record Level:** collectionID: Western Foundation of Vertebrate Zoology; collectionCode: WFVZ
- **Type status:** Other material. **Occurrence:** catalogNumber: WFVZ-12980; occurrenceID: WFVZ12980; **Taxon:** scientificName: Lesbia nuna; **Location:** country: Colombia; stateProvince: Narińo; municipality: Mallama; locality: El Guabo; decimalLatitude: 1.116600; decimalLongitude: -77.816600; **Record Level:** collectionID: Western Foundation of Vertebrate Zoology; collectionCode: WFVZ
- **Type status:** Other material. **Occurrence:** catalogNumber: ICN-7825; occurrenceID: ICN7825; **Taxon:** scientificName: Lesbia nuna; **Location:** country: Colombia; stateProvince: Narińo; municipality: Pasto; locality: Matituy; decimalLatitude: 1.350000; decimalLongitude: -77.333300; **Record Level:** collectionID: Instituto de Ciencias Naturales; collectionCode: ICN
- **Type status:** Other material. **Occurrence:** catalogNumber: MCBM-43; occurrenceID: MCBM43; **Taxon:** scientificName: Lesbia nuna; **Location:** country: Colombia; stateProvince: Narińo; municipality: Pasto; locality: Pasto; decimalLatitude: 1.216600; decimalLongitude: -77.266600; **Record Level:** collectionID: Museo Madre Caridad Brader Zahner; collectionCode: MCBM
- **Type status:** Other material. **Occurrence:** associatedReferences: Willis & Schuchmann (1993); occurrenceID: WillisANDSchuchmann199301; **Taxon:** scientificName: Lesbia nuna; **Location:** country: Colombia; stateProvince: Narińo; municipality: Ricaurte; locality: La Planada; decimalLatitude: 1.221536; decimalLongitude: -77.983802
- **Type status:** Other material. **Occurrence:** catalogNumber: ROM-94757; occurrenceID: ROM94757; **Taxon:** scientificName: Lesbia nuna; **Location:** country: Colombia; stateProvince: Narińo; municipality: Túquerres; locality: Guachaves; decimalLatitude: 1.216600; decimalLongitude: -77.683300; **Record Level:** collectionID: Royal Ontario Museum; collectionCode: ROM
- **Type status:** Other material. **Occurrence:** catalogNumber: ROM-78502; occurrenceID: ROM78502; **Taxon:** scientificName: Lesbia nuna; **Location:** country: Colombia; stateProvince: Putumayo; municipality: San Francisco; locality: Sibundoy; decimalLatitude: 1.183300; decimalLongitude: -76.916600; **Record Level:** collectionID: Royal Ontario Museum; collectionCode: ROM
- **Type status:** Other material. **Occurrence:** occurrenceRemarks: Excluded from ENMs; associatedReferences: Losada-Prado et al. (2005); occurrenceID: LosadaPradoETAL200501; **Taxon:** scientificName: Lesbia nuna; **Location:** country: Colombia; stateProvince: Tolima; municipality: Cajamarca; locality: río Anaime; decimalLatitude: 4.439100; decimalLongitude: -75.423600
- **Type status:** Other material. **Occurrence:** associatedReferences: Losada-Prado et al. (2005); occurrenceID: LosadaPradoETAL200502; **Taxon:** scientificName: Lesbia nuna; **Location:** country: Colombia; stateProvince: Tolima; municipality: Ibagué; locality: río Combeima; decimalLatitude: 4.316600; decimalLongitude: -75.150000

#### Description

Appendix 1. List of localities where *L. nuna* has been recorded in Colombia and included in the modelling process. Coordinates are presented in the decimal degree system. Acronyms: (AMNH) American Museum of Natural History; (ANS) Academy of Natural Sciences, Philadelphia; (DMNH) Delaware Natural History Museum; (FMNH) Field Museum of Natural History; (IAvH) Instituto Alexander von Humboldt; (ICN) Instituto de Ciencias Naturales; (INCIVA) Instituto Vallecaucano de Investigaciones; (IBC) Internet Bird Collection (http://ibc.lynxeds.com/photo/green-tailed-trainbearer-lesbia-nuna/male-feeding-blackberry-flower); (LACM) Natural History Museum of Los Angeles County; (MCBM) Museo Madre Caridad Brader Zahner; (MCZ) Museum of Comparative Zoology, Harvard University; (MHNCSJ) Museo de Historia Natural, Colegio San José; (MHN-UCC) Universidad del Cauca; (MLS) Universidad de la Salle; (MVZ) Museum of Vertebrate Zoology, University of California, Berkeley; (NHM) Natural History Museum, London; (RMNH) Nationaal Natuurhistorisch Museum; (RMNH) Naturalis Biodiversity Center; (ROM) Royal Ontario Museum; (UNIANDES) Universidad de los Andes; (USNM) National Museum of Natural History; (WFVZ) Western Foundation of Vertebrate Zoology; (XC) Xeno-Canto (www.xeno-canto.org); (YPM) Yale Peabody Museum.

## Analysis

### Occurrence dataset

**Figure 1.**
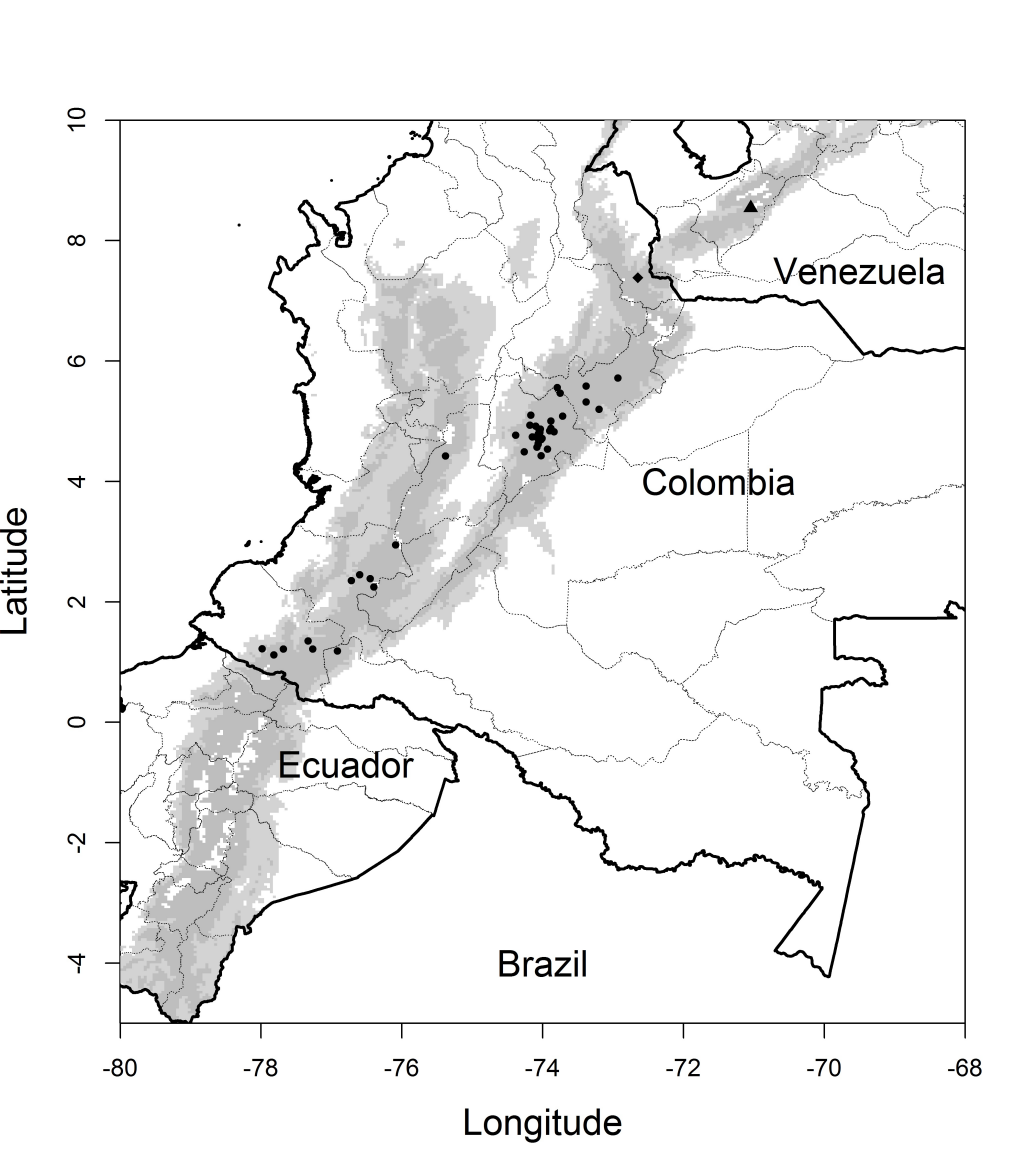
Map of northern Andes showing: (1) areas from 700 m to 1700 m a.s.l. (lighter grey); (2) areas from 1700 m to 3800 m a.s.l. (darker grey); (3) recording localities of *L. n. gouldii* used in the authors' ENMs analyses (solid circles); (4) "Pamplona" in Norte de Santander Department (solid diamond); (5) Bolívar Peak (= “El Picácho de la Colúna” according to Goering 1958) in whose vicinity the “Venezuelan” specimen of *L. n. gouldii* would have been collected (solid triangle).

The record catalogue number 1888.7.25.185 (Natural History Museum, London) was excluded given its obscure origin (Zimmer 1951). All specimens from “El Guabo” (Nariño) were considered as belonging to the homonymous locality in the Mallama municipality. Information was gathered on 115 records of *L. n. gouldii*, which were reduced to 46 localities after removing duplicates and redundant records and reduced to 42 occurrence data points after removing the ~10% (four) most spatially autocorrelated ones, without counting Pamplona in Norte de Santander Department (Fig. 1). The dataset from the web-based repositories eBird Basic Dataset (2017),(Sullivan et al. 2009) and gbif.org (2017) consisted of 699 sightings (Appendix 2).

### Environmental variables

A correlation matrix (Table 1) indicated three groups of highly correlated variables. The first two were integrated by temperature-based variables: (1) Bio01, Bio05, Bio06, Bio08, Bio09, Bio10 and Bio11, which were, in turn, highly correlated with elevation and (2) the pair Bio02 and Bio07. The third group was a set of the precipitation-based variables Bio12, Bio13, Bio14, Bio16, Bio 17 and Bio 19. Although Bio12 (annual precipitation) and Bio14 (precipitation in the driest month) belonged to the same group, they were not highly correlated with each other, so both were retained for further analyses, in addition to elevation and Bio07 (annual temperature range). Correlations of variables Bio03 (isothermality), Bio04 (temperature seasonality), Bio15 (precipitation seasonality), Bio18 (precipitation in the warmest quarter), soil and geology were below 0.75 in all cases and thus included in the modelling process.

**Figure 2.**
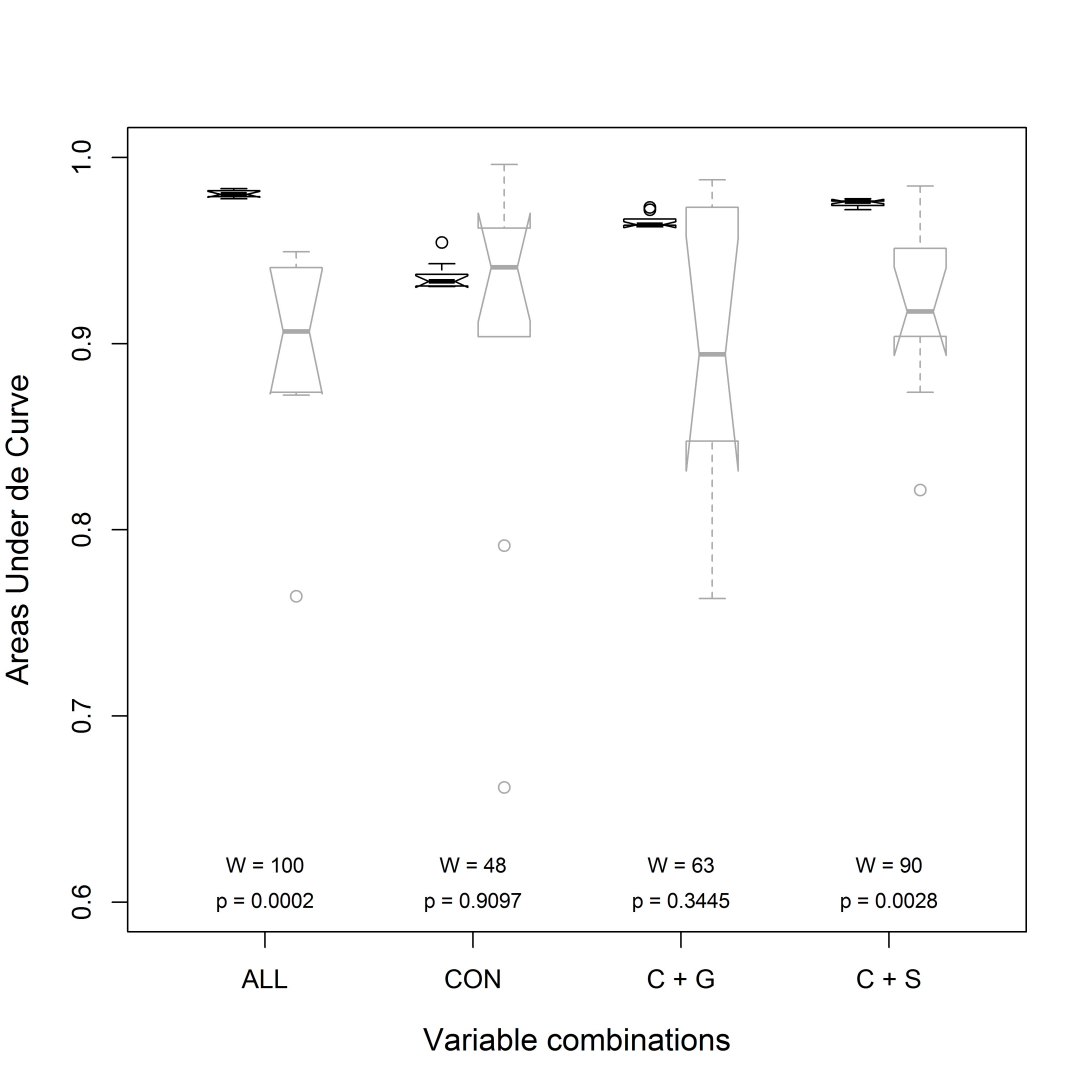
Comparison of the areas under the curves for both training (black) and test (grey) of the ENMs using the 42 localities where *Lesbia nuna* has been recorded in Colombia (excluding "Pamplona" and the four most highly autocorrelated localities) for each of the four environmental variable combinations: continuous variables alone (= climate and elevation, CON), continuous variables and geology (C+G), continuous variables and soil (C+S) and the combination of all (ALL). “W” and “p” are, respectively, the values of the statistics and the probability of Wilcoxon rank sum tests.

All variables showed some degree of spatial autocorrelation. Moran’s I coefficients ranged from 0.533 (Bio03) to 0.086 (elevation), averaging 0.290. After removing four localities, these values were substantially reduced to a range between 0.465 (Bio03) and 0.002 (elevation), averaging 0.207. The omission rate on test samples was higher than the predicted omission rate when incorporating soil information. A similar pattern is observed when comparing the areas under the curves amongst the different combination of variables (Fig. 2): the AUC_Train_ is statistically higher than AUC_Test_ when incorporating soil in the analyses. For instance, removing localities and incorporating soil information reduced the influence of the spatial autocorrelation on the models, improving the independence between test and training data.

Summary statistics for variables used for ENMs are shown in Table 2. Bioclimatic variables based on temperature showed similar patterns at recording points in Colombia when compared with locations within the same elevational range in the Venezuelan Andes. In contrast, variables involving precipitation showed substantial differences between both areas.

Occurrence points in Colombia are found in four of the geologic provinces (sensu Schenk et al. 1999): (1) West-Central Cordillera, (2) Cauca Basin, (3) Eastern Cordillera and (4) Llanos Basin. In particular, 35.7% of occurrence points fall in areas of Cretaceous origin, 31.0% during the Tertiary, 11.9% during the Quaternary and 21.4% during other periods. In contrast, the whole area within the elevational range of *L. nuna* belongs to just one of those provinces, the Perijá-Venezuelan Coastal Ranges, where 24.7% of records fall in areas originating in the Precambrian undifferentiated, 20.6% in Paleozoic metamorphics, 6.4% in Cretaceous and 48.3% in other periods.

In Colombia, soils were mostly Leptosols (40.5% of the points), followed by Acrisols (23.8%), various Cambisols (21.4%), various Phaeozems (11.9%) and Luvisols (2.4%) while, in Venezuela, various Cambisols predominated (61.7%), followed by Leptosols (19.0%), Luvisols (10.6%), Ferralsols (5.5%), Solonetz (2.5%) and Arenosols (0.5%)i.e., weakly developed soils whose development has been limited by landscape instability (Beek and Bramao 1969, de Castro Portes et al. 2016).

The relative contribution of each environmental variable to the different models is shown in Table 3. Elevation had the highest contribution in the CON model and the corresponding permutation importance (i.e. the jackknife significance test) indicates heavy dependence of this model on that variable. Geology had the highest contribution in the case of the C+G model, but the corresponding permutation importance indicates that this model depends more on elevation. Finally, soil had the highest contribution in the case of the C+S and ALL models.

### Threshold selection

**Figure 3.**
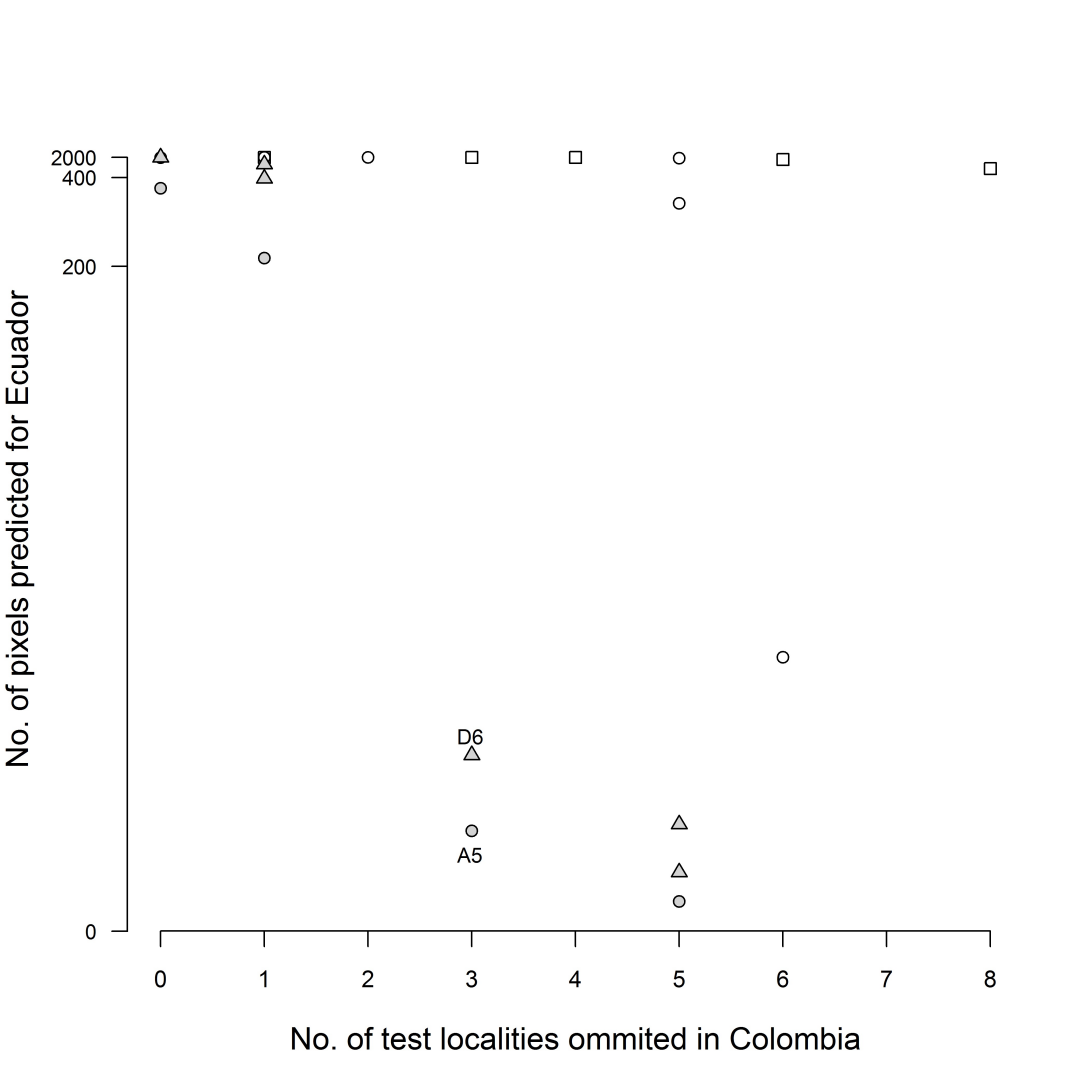
Number of *Lesbia nuna* test localities omitted in Colombia, contrasted against the number of pixels predicted for Ecuador. ● ALL models, ▲C+S , ○ CON, □ C+G, combined with seven thresholds. A5 refers to ALL model + Equal training sensitivity and specificity logistic threshold and D6 refers to C+S + Maximum training sensitivity plus specificity logistic threshold.

All combinations of variables, especially CONs, predicted suitable areas in Ecuador, false negatives in Colombia and suitable areas in Venezuela, when using given thresholds. Two combinations of variables and thresholds performed differentially better (Fig. 3): ALL + Equal training sensitivity and specificity logistic threshold and C+S + Maximum training sensitivity plus specificity logistic threshold.

These combinations only predicted three false negatives in the case of Colombia (Fig. 4) where the suitable habitat for this hummingbird subspecies consists of a relatively wide area on the Cundiboyacense altiplano and smaller to scattered areas in both the Massif of Huaca and along the Central Cordillera up to central Antioquia. These combinations did not predict suitable areas in Norte de Santander which excludes Pamplona but predicted small suitable areas in Ecuador of 17 or 29 (out of a total of 34) pixels in two small patches at Tungurahua and Loja. These combinations also predicted very small suitable areas for *L. n. gouldii* in Venezuela: 4 and 18 (out of a total of 19) pixels located in eastern-central Táchira and a couple in the westernmost portion of Mérida State i.e. these models did not predict the “Sierra Nevada” as a suitable habitat.

Similar results were achieved when including Pamplona in the ENMs with three main differences: (1) only one combination performed differentially better (C+S + X10 percentile training presence logistic threshold), (2) fewer, small and scattered patches of suitable areas predicted in Norte de Santander and (3) the same, but smaller, two patches of suitable areas predicted for Ecuador. This model also predicted some suitable areas for Venezuela similar to, but smaller than those described in the previous paragraph, excluding again “Sierra Nevada, Merida”.

## Discussion

### Model selection

Habitat suitability for *L. n. gouldii* under current conditions was predicted using bioclimatic variables, elevation, information on geology and soil, as well as data available on the distribution of this hummingbird. However, the highest omission rate on test samples compared to the predicted omission rate and the statistically significant differences between the AUC_Train_ and AUC_Test_ strongly indicated that models including soil information should be preferred in this case. Moreover, the combination of few omissions of test localities in Colombia and the small area predicted for Ecuador, highlight the convenience of applying the Equal training sensitivity and specificity logistic threshold, as well as the Maximum training sensitivity plus specificity logistic thresholds. The authors' conclusions are based on these modelling conditions.

These predictions fitted almost exactly to the range reported by the independent datasets consulted (Fig. 4), reinforcing the premise that this model consistently retrieves the actual distribution range of *L. n. gouldii* in the northern Andes. This included areas as far as the northernmost portion of the Central Cordillera in Colombia whose validity is corroborated by the visual record from Entrerrios (CORANTIOQUIA, catalogue number 4743-5615). In contrast to Meyer de Schauensee (1982), these predictions clearly excluded the whole Norte de Santander Department. Of course, “accidentals” have been largely recognised in literature (e.g. Grinnell 1922), but stable populations are more likely to be absent from Norte de Santander as reinforced by the lack of further records from eBird (Sullivan et al. 2009, eBird Basic Dataset 2017) and gbif.org (2017).

### Contribution of variables

Climate variability characterised the Holocene (11,500 BP to the present), with several periods of significant rapid climate change of polar cooling, tropical variation of moisture and major atmospheric circulation changes (Mayewski et al. 2004, Polissar et al. 2013). For example, temperature lowered to −3.2 ± 1.4°C and precipitation increased *ca.* 20% between 1250 and 1810 CE in the Venezuelan Andes, promoting four glacial advances (Polissar et al. 2006) with corresponding changes in the biota. For example, Rull et al. (1987) and Rull and Schubert (1989) postulate that the Little Ice Age caused the lowering of vegetation belts in the Venezuelan Andes during the 15-16th centuries. To succeed in dealing with such environmental uncertainty, organisms would have to follow certain strategies, such as: (1) a conservative bet-hedging that minimises their fitness variance across all possible environmental conditions (Starrfelt and Kokko 2012), (2) a diversification bet-hedging that takes advantage of alternative environmental scenarios in a probabilistic fashion (Starrfelt and Kokko 2012) and (3) an adaptive tracking in which the environmental variation results in correlated variation in mean population traits as natural selection favours different phenotypes over evolutionary time (Cleland et al. 2007). That is, the characteristic climatic unpredictability of most of the “life span” of *L. n. gouldii* may have kept or promoted a plasticity with respect to the tolerance of climatic conditions, as suggested by its wide elevational range and the low to zero contribution of the bioclimatic variables to ALL and C+S models.

Conversely, soil and geology are more stable features and were the most important in *L. n. gouldii* than any other variable when included in the modelling processes. Bedrock geochemistry (Hahm et al. 2014) and soil properties (Aragão et al. 2009, Honorio Coronado et al. 2009, Unger et al. 2012, Muenchow et al. 2013, Arellano et al. 2014) influence plant species distribution, composition, productivity and structure which, in turn, influence animal species abundance and composition (Jankowski et al. 2012, Pomara et al. 2012) and ultimately animal distribution (Peres 2008, Beja et al. 2010), including very mobile ones such as bats (Ramos Pereira et al. 2009).

In South American lowlands, where the effect of physical barriers is expected to be low, a broad range of evidence from plants (Kreft et al. 2004), arthropods (Sigrist and de Carvalho 2009), amphibians (Symula et al. 2003), reptiles (Vanzolini 1988, Sigrist and de Carvalho 2009), birds (Haffer 1987, Prum 1988, Bates et al. 1998) and mammals (da Silva and Oren 1996, Patton and da Silva 1998) indicates an unevenly distributed biodiversity with areas holding high endemism and unique biotas, whose origins, history and ecological mechanisms are debated (Tuomisto and Ruokolainen 1997, Bush 2005, Haffer 2008) but are consistent with the hypothesis that current patterns of biotic distribution in the Amazon basin are based on edaphic differences (Salo 1987).

Moreover, most of the articles cited in the previous paragraph and (up to a point) one of the currently accepted ecoregional divisions of South America (Olson et al. 2001), point to the Putumayo River as the southernmost limit, similar to that reported by Ramoni-Perazzi et al. (2012) for the mid-elevation-ranging phyllostomid bat *Artibeus amplus*. Thus, these results suggest the presence of edaphic differences acting as ecological barriers to *L. n. gouldii* as a possible bottom-up effect of soil properties on the distribution of this hummingbird.

Adaptation to local environmental conditions is a primary driver for morphological evolution and speciation (Schluter 2000, Schluter 2001, Levin 2003). This important stage in the speciation process is identifiable through examination of the ecological niches, thus strengthening support for species delimitation (Funk et al. 2006, Kozak and Wiens 2006, Rissler et al. 2007, Kozak et al. 2008, Wielstra et al. 2012). In the Andes, speciation has been promoted by abundant orographic barriers and step elevational gradients (Vuilleumier 1969, Vuilleumier 1984, Simpson Vuilleumier 1971, Fjeldså 1992) as well as temporary isolation by glacial cycles (e.g. Vuilleumier and Simberloff 1980, Fjeldså 1992, Heindl and Schuchmann 1998, García-Moreno and Fjeldså 2000). In the case of *L. n. gouldii*, this probably resulted from a population isolated in suitable areas of the Cauca and Magdalena valleys during the LGM evolving in isolation and migrating upslope to suitable areas during the temperature rise in the Holocene. In this context and reinforced by the lack of evident physical barriers separating *L. n. gouldii* from its neighbour *L. n. gracilis*, these results are in favour of the proposal of Weller and Schuchmann (2004) to split *L. gouldii* from *L. nuna*.

### *Lesbia nuna gouldii* in Venezuela

The lack of further *L. n. gouldii* records for Venezuela can be analysed through three postulates. First, trochilids include long-distance and elevational migrants, acknowledged for their ability to travel long distances. Furthermore, most Andean hummingbird species have patchy distribution patterns including prominent cases such as *Eriocnemis luciani*, whose population in Ecuador and the extreme southwestern Colombia is separated by a gap of *ca.* 1100 km in Eastern Cordillera from a population in the Venezuelan Andes (Schuchmann et al. 2001). For instance, *L. nuna* could have reached the Mérida Cordillera during the LGM and then gone locally extinct simply by chance as shown for insular species on oceanic islands (MacArthur and Wilson 1967, Brown 1978). In fact, Venezuelan Andes are smaller than their Colombian counterparts, hence the lower number of avian species observed in the former. Mapping the elevation of the northern Andes from 700 m a.s.l. (the lowest elevation expected for *L. nuna* during the LGM) and upwards, shows a continuous belt from Colombia to Venezuela which is interrupted nowadays only by the Táchira depression (Fig. 1) whose efficacy as a biogeographic barrier varies from one taxonomic group to another (Gutiérrez et al. 2015). Therefore, *L. n. gouldii* should range up to at least the Tama Massif, but this area was excluded by the authors' ENMs and is not supported by collecting/recording information.

Second, *L. n. gouldii* could have occurred in the Venezuelan Andes until historical times but the dynamics of habitat transformation on both sides of the Colombian-Venezuelan border led to its extinction in the latter country. However, similar considerations should be made in this case as in the previous paragraph. Moreover, (1) the “Venezuelan” locality is a well-preserved area minimally impacted by human activities and (2) in Colombia, this hummingbird has been considered “fairly common” (Restall et al. 2006) and some eBird Basic Dataset (2017) records have been made in parks in Bogotá city, highlighting the tolerance of this species to habitat transformation.

Third, this hummingbird was never established in the Venezuelan Andes, as indicated by these analyses. Available data indicate a substantial variation in precipitation and temperature patterns with latitude along the tropical Andes since the LGM and thus regions sharing synchronous changes during one period could be asynchronous during another Bush et al. (2011). For example, from *ca.* 8000 BP to the present, the climate of northern South America has been influenced by both El Niño-Southern Oscillation and the Intertropical Convergence Zone, but during the Younger Dryas and the early Holocene, western and eastern regions were differentially influenced by these climatic phenomena (Muñoz et al. 2017). In fact, in their GIS-based vegetation map of the world at the time of the LGM, Ray and Adams (2001) indicated differences between the drier Venezuelan Andes, occupied by semi-desert to the northwest and grasslands to the southeast and the wetter Colombian Andes, occupied by a complex topographic mosaic of forests, grasslands and montane deserts. Thus, despite its being very likely that the dispersal capabilities of these hummingbirds could have led them to spread to the Venezuelan Andes at some point in past times, differences in the evolution of environmental conditions could have prevented them establishing there permanently.

Moreover, the information in Fig. 4 strongly suggests that the northeastern limit of *L. n. gouldii* coincides with the Chicamocha Canyon. The origins of the Eastern Cordillera have been debated (Taboada et al. 2000) but widely recognised as resulting from an asynchronous and spatially heterogeneous process, as evidenced by the four “Massifs”: Garzón, Quetame, Floresta and Santander (Restrepo and Toussaint 1988, Case et al. 1991, Restrepo-Pace et al. 1997). From a geological point of view, Mérida and the Eastern Cordilleras are separated by the NW–SE trending Santander Massif and the southern termination of the left-lateral strike-slip Santa Marta-Bucaramanga fault (Audemard M 2003), the system to which the Chicamocha Canyon belongs. From a biogeographical point of view, the Chicamocha Canyon represents a barrier or a discontinuity for several taxa (Cuatrecasas 1979, Cuervo 2013). For example, the frog genus *Rheobates* (Aromobatidae) has a highly supported genetic discontinuity corresponding roughly to a split centred on the Chicamocha Canyon (Muñoz-Ortiz et al. 2014). Amongst birds, within the Long-tailed Antbird species complex, *Drymophila caudata* (Thamnophilidae), characterised by its large range in both latitude (from northern Venezuela to Bolivia) and elevation (800 to 3150 m), the Chicamocha Canyon is the barrier between *D. caudata*, distributed to the southwest and its vicariant *D. klagesi*, found in the northeast (Isler et al. 2012). In addition, the subspecies of the Pale-bellied Tapaculo, *Scytalopus griseicollis* (Rhinocryptidae), ranging between 2000 and 3900 m, are separated from each other by the system of the Chicamocha Canyon and the Horta-Opón valley (Avendaño and Donegan 2015).

The possibility of an "accidental" status of the "Venezuelan" specimen is also possible, but a clue in this respect can be obtained directly from the alleged collector: Christian Anton Goering. According to Sclater and Salvin (1868), this German ornithologist, painter and explorer, arrived at Carúpano dock, Sucre, on 30th November 1866 and stayed in Venezuela until 1874 (Sclater and Salvin 1875). He was commissioned by the Zoological Society of London to collect specimens of the Venezuelan fauna for the British Museum, arriving in “Merida by way of the Lake of Maracaibo and Zuliar on 5th April, 1869” and “Leaving Merida on 30th of October, 1869, Mr. Goering set out to return by land to Puerto Cabello, intending to collect *en route*. But reaching Carache, a revolution broke out, which rendered it necessary for him to retreat to the Lake of Maracaibo and so by sea to La Guayra” (Sclater and Salvin 1870). According to Sclater and Salvin (1875), who indicated no precise date but “Previously to his return to Europe last year”, Herr Goering performed a second trip to Mérida when he ascended the Sierra Nevada to (at least) “an altitude of 10,000 feet”, when he would have collected the “Venezuelan” specimen of *L. nuna*. Then, “After leaving Merida, on his last journey, Mr. Goering traversed the line of the Andes to San Cristoval, in the Province of Tachira, on the frontiers of Columbia” (Sclater and Salvin 1875) without indicating how Herr Goering returned to Caracas.

However, the journey depicted in the previous paragraph contrasts with many of the details narrated by Herr Goering himself in his book published in Leipzig in 1893, translated into Spanish by M. L. de Blay and published by Universidad de Los Andes, Mérida, Venezuela, in 1958, thisbeing the version consulted by the authors. According to Goering (1958), he performed only one journey to the Venezuelan Andes (not two as indicated in the previous paragraph), when besides exploring the surroundings of Mérida city, he performed three round-trip expeditions: (1) towards Torondoy, southern part of the Lake of Maracaibo Basin, crossing the páramo of Sierra de La Culata at Mucuchíes; (2) towards Cúcuta, in Norte de Santander Department, Colombia, crossing the Mocotíes valley and the Táchira State; and (3) towards Sierra Nevada in his attempt to climb “El Picácho de la Colúna” (= “Pico La Columna”, or “Pico Bolívar” since 1925) in June. Then Goering left Mérida intending, as indicated by Sclater and Salvin (1870), to return by land to Puerto Cabello but a revolution forced him to retreat to the Lake of Maracaibo, where he lost part of the specimens collected and then he continued by sea to Puerto Cabello (not Caracas) where he sent the surviving specimens to England. Thereafter, Goering (1958) spent the remaining time in Venezuela surveying Valencia, the Llanos, Guacara and Caracas, where he witnessed three days of fighting in 1870 and where he associated with local and foreign personalities, such as Mr. James Mudie Spence, who promoted an artistic exhibition about the middle of 1872 andwhere Goering showed some 50 drawings and paintings, some of which were used by Spence (1878). So, according to Goering himself, he was not in Sierra Nevada in 1873, when the “Venezuelan” specimen of *L. nuna* would have been collected.

Moreover, the book of Goering (1958) contains many remarks on birds collected by him, because of their beauty, as they represented some novelty, as they were new to him etc. With regards to his surveys around Mérida city, he wrote “Characteristic hummingbird species of these upper forests are Heliangelus Spenci, Bourciera Conradi […] and the Sword-bill Hummingbird (Docimastes ensifer)”. In the case of his ascent to Sierra Nevada, an enterprise narrated in an entire chapter of his book, Herr Goering offered a short list of the birds collected: “Anthus bogotensis, *Phrygillus unicolor*, *Serpophaga cyanea*, *Ochtoëca superciliosa*, *Turdus gigas*, […] *Querquedula* andium […]. At an elevation of 3500 m, I found a new species of parrot, the *Conurus rhodocephalus* along with a black and white Water Thrush (Cinclus leuconotus). In the shrub area also occurs Stegnolaema Montagnii.”

**Figure 5.**
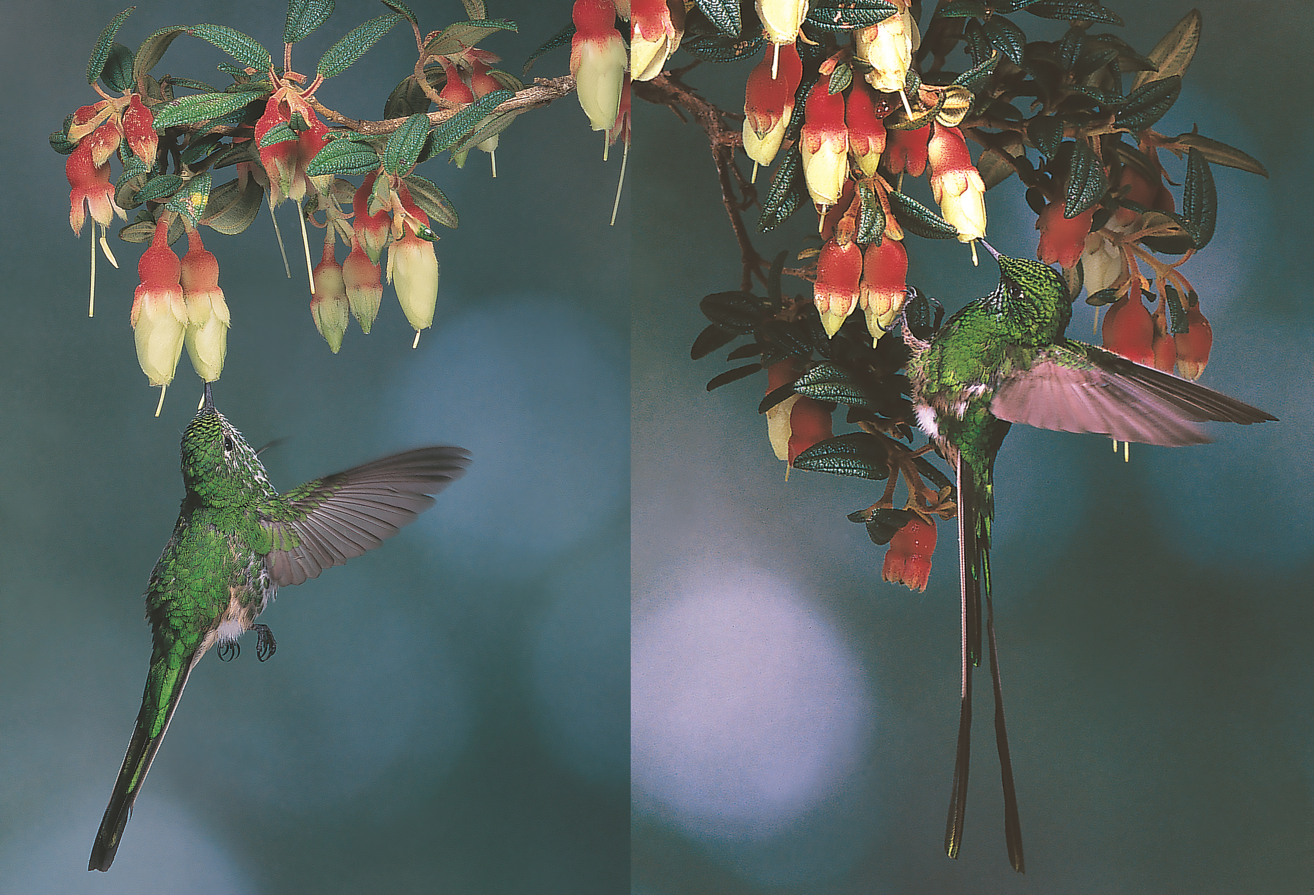
Female (left) and male (right) Green-tailed Trainbearers (*Lesbia nuna*), from Volcan Pichincha, Ecuador. Photos: Dr. Luis Mazariegos.

Therefore, it is striking that a spectacular species like *L. nuna* (Fig. 5), whose beauty should have added a note of colour (because "*The local fauna found here* [at Sierra Nevada] *is well adapted* [morphologically] t*o the landscape; no colorful birds nor insects can be observed*") and which would have been new to Herr Goering, representing a new record for Venezuela and an interesting record of this species, was excluded from his report.

All this suggests that the “Venezuelan” specimen was simply a case of mislabelling, "*perhaps from the large collections of these birds* [hummingbirds] *that are constantly being forwarded from the vicinity of Bogotá*" (Sclater and Salvin 1870).

In conclusion, it is very unlikely that the range of *L. nuna* extended to Venezuela or that it even occurred in the country as an accidental visitor. In consequence, this species should be removed from the Venezuelan bird list.

## Supplementary Material

XML Treatment for Lesbia nuna

## Figures and Tables

**Figure 4a.**
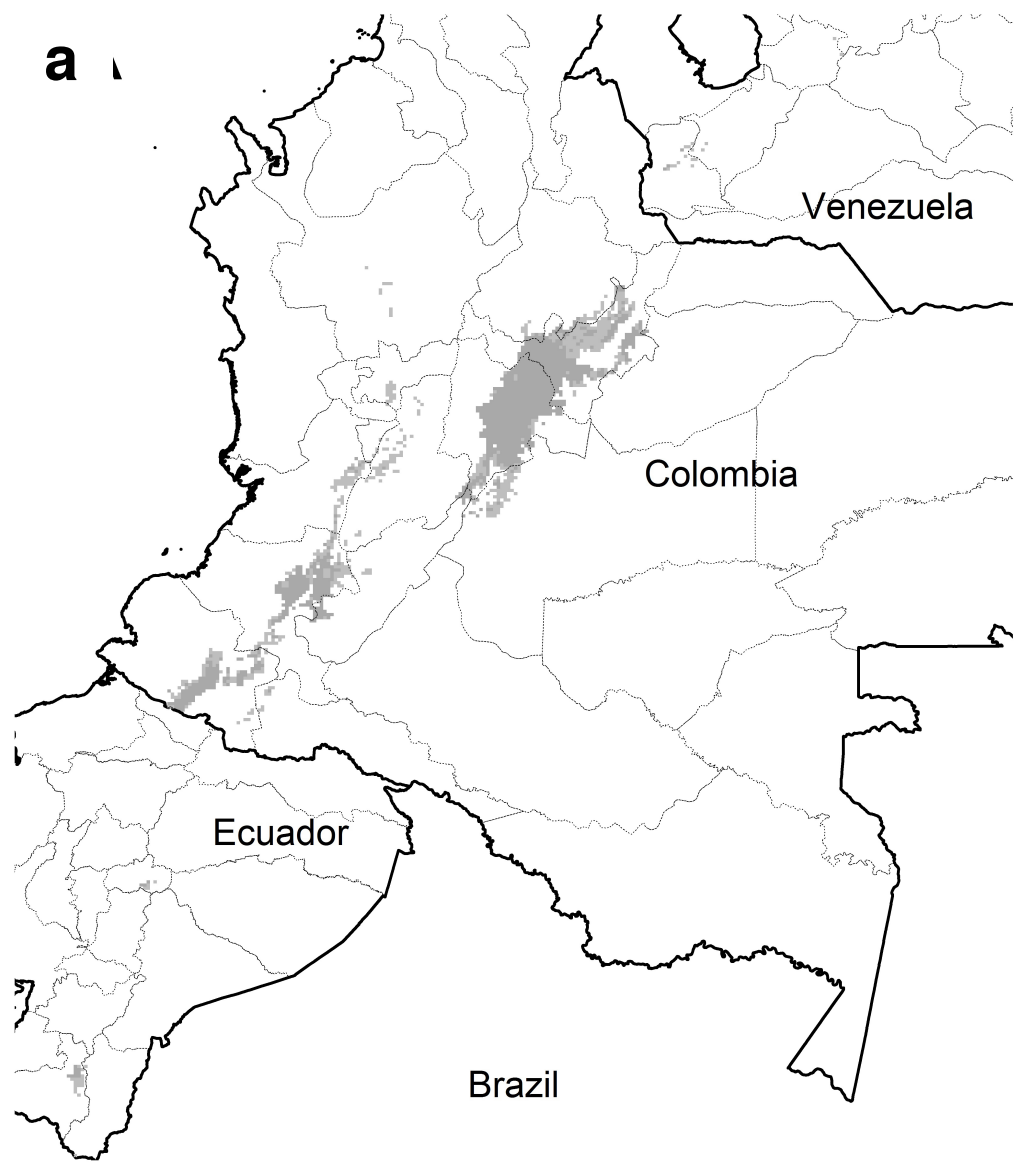
Habitat predicted by ALL model + Equal training sensitivity and specificity logistic threshold. Lighter grey: areas predicted by only one of these parameter combinations. Darker grey: areas predicted by both parameter combinations

**Figure 4b.**
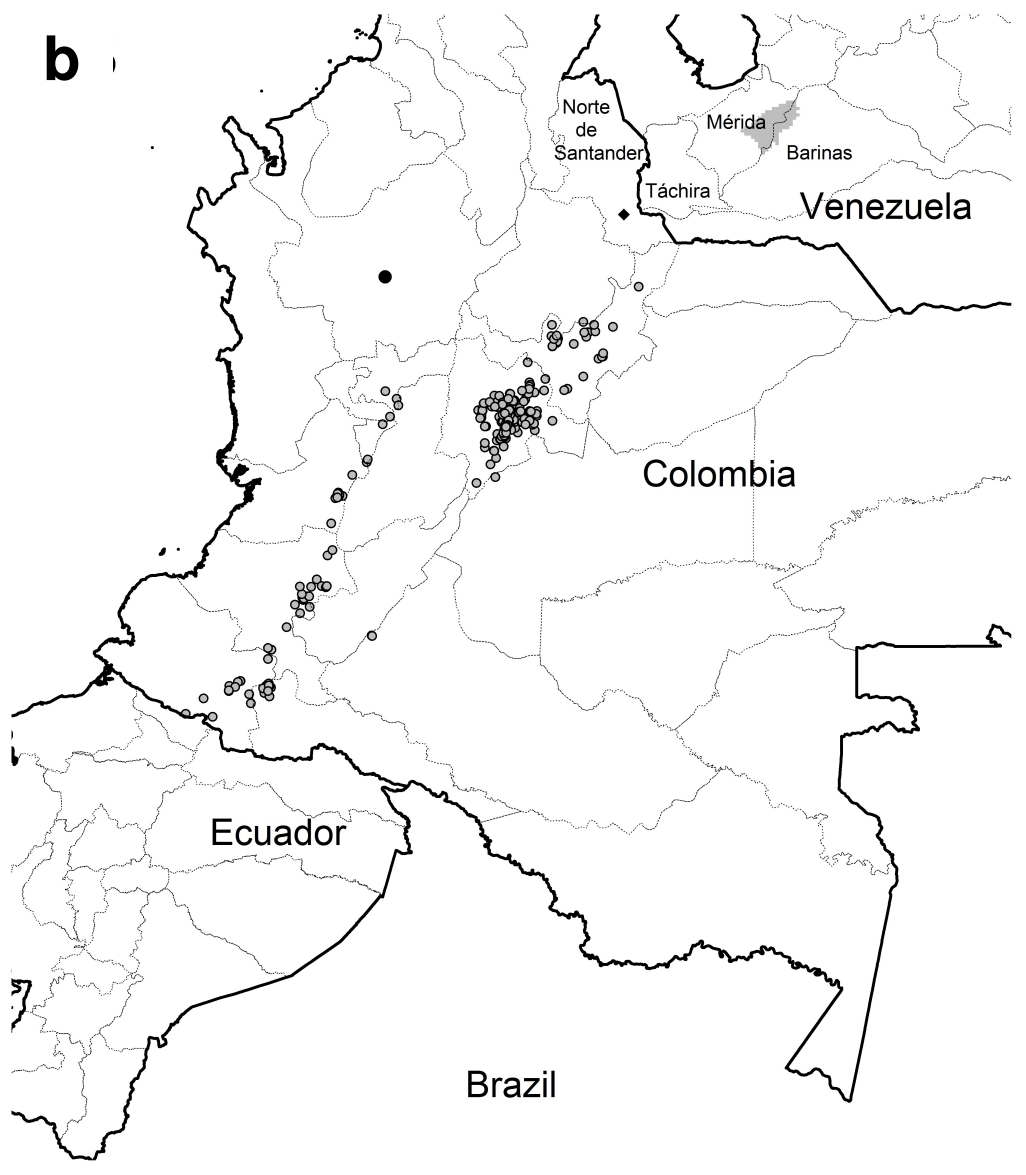
C+S + Maximum training sensitivity plus specificity logistic threshold Grey circles: localities where *L. nuna* has been observed in Colombia as listed in Appendix 2 ● Entrerrios locality, Antioquia Department (CORANTIOQUIA, catalogue number 4743-5615). ♦ Pamplona, Norte de Santander. Grey area between Mérida and Barinas States in Venezuela corresponds to the approximate extent of the Sierra Nevada.

**Table 1.** Pearson's correlations between continuous environmental variables, polyserial correlations between continuous and categorical environmental variables and polychoric correlations between the categorical environmental variables for the record localities of *Lesbia nuna* in Colombia. Continuous variables: Elev, elevation; Bio01, annual mean temperature; Bio02, mean monthly temperature range; Bio03, isothermality; Bio04, temperature seasonality; Bio05, max temperature of the warmest month; Bio06, min temperature of the coldest month; Bio07, temperature annual range; Bio08, mean temperature of the wettest quarter; Bio09, mean temperature of the driest quarter; Bio10, mean temperature of the warmest quarter; Bio11, mean temperature of the coldest quarter; Bio12, annual precipitation; Bio13, precipitation of the wettest month; Bio14, precipitation of the driest month; Bio15, precipitation seasonality; Bio16, precipitation of the wettest quarter; Bio17, precipitation of the driest quarter; Bio18, precipitation of warmest quarter; Bio19, precipitation of the coldest quarter. Categorical variables: Soil and Geol, geology.

	Bio01	Bio02	Bio03	Bio04	Bio05	Bio06	Bio07	Bio08	Bio09	Bio10	Bio11	Bio12	Bio13	Bio14	Bio15	Bio16	Bio17	Bio18	Bio19	Soil	Geol
Elev	-0.99	-0.41	-0.29	-0.07	-0.96	-0.97	-0.10	-0.98	-0.99	-0.98	-0.99	-0.34	-0.44	0.07	-0.47	-0.44	0.04	0.04	-0.54	-0.28	-0.18
Bio01	-	0.40	0.22	0.14	0.98	0.97	0.14	1.00	1.00	1.00	1.00	0.26	0.36	-0.17	0.52	0.37	-0.15	-0.09	0.48	0.20	0.12
Bio02		-	-0.23	0.03	0.54	0.21	0.83	0.42	0.40	0.40	0.41	-0.19	-0.04	-0.27	0.35	-0.06	-0.30	-0.55	0.03	-0.20	0.16
Bio03			-	-0.44	0.09	0.40	-0.74	0.20	0.22	0.18	0.24	0.61	0.54	0.58	-0.12	0.54	0.59	0.60	0.27	0.58	0.62
Bio04				-	0.22	0.11	0.27	0.12	0.18	0.20	0.08	-0.01	0.04	-0.18	0.24	0.06	-0.20	-0.25	0.20	-0.22	-0.40
Bio05					-	0.92	0.31	0.98	0.98	0.98	0.98	0.20	0.33	-0.24	0.57	0.33	-0.22	-0.22	0.47	0.11	0.07
Bio06						-	-0.10	0.96	0.97	0.96	0.96	0.42	0.50	-0.02	0.47	0.50	0.01	0.07	0.56	0.31	0.17
Bio07							-	0.16	0.13	0.16	0.14	-0.50	-0.36	-0.53	0.29	-0.37	-0.56	-0.72	-0.16	-0.48	-0.24
Bio08								-	0.99	0.99	1.00	0.22	0.32	-0.19	0.49	0.32	-0.17	-0.10	0.44	0.18	0.11
Bio09									-	1.00	0.99	0.29	0.39	-0.14	0.51	0.40	-0.12	-0.09	0.52	0.21	0.11
Bio10										-	0.99	0.24	0.35	-0.20	0.54	0.35	-0.18	-0.11	0.47	0.17	0.09
Bio11											-	0.25	0.35	-0.17	0.52	0.36	-0.15	-0.09	0.46	0.20	0.14
Bio12												-	0.95	0.72	-0.09	0.97	0.77	0.61	0.86	0.56	0.23
Bio13													-	0.58	0.16	0.99	0.63	0.42	0.89	0.49	0.23
Bio14														-	-0.60	0.57	0.98	0.67	0.44	0.43	0.17
Bio15															-	0.12	-0.58	-0.49	0.12	-0.19	0.05
Bio16																-	0.62	0.44	0.91	0.53	0.24
Bio17																	-	0.73	0.49	0.44	0.16
Bio18																		-	0.26	0.34	0.09
Bio19																			-	0.45	0.02
Soil																				-	0.46

**Table 2.** Summary statistics for explanatory variables used in ENMs for the 42 localities where *Lesbia nuna* has been recorded in Colombia and for the same elevational range in the Venezuelan Andes. In the categorical variables, “n” refers to the number of localities (Colombia) or the number of pixels (Venezuela). In Geology, codes correspond to those provided by layer GEO6EXP_ID.

**Variable**	**Colombia**			**Venezuela**		
Continuous	Median	Min	Max	Median	Min	Max
Elev	2614.0	1654.0	3522.0	2263.0	1654.0	3522.0
Bio03	81.0	75.0	91.0	81.0	75.0	84.0
Bio04	303.5	185.0	640.0	428.0	339.0	608.0
Bio07	113.0	98.0	141.0	137.0	110.0	149.0
Bio12	991.5	772.0	2285.0	1021.0	706.0	1478.0
Bio14	35.0	20.0	123.0	22.0	8.0	38.0
Bio15	40.0	25.0	60.0	49.0	37.0	68.0
Bio18	290.5	153.0	685.0	310.0	198.0	503.0
Nominal	Code	*n*	%	Code	*n*	%
Soil	16039	17	40.5	27614	172	30.8
	16047	10	23.8	27615	172	30.8
	16017	4	9.5	27616	107	19.1
	16015	4	9.5	27621	59	10.6
	Others(04)	7	16.7	Others (04)	49	8.8
Geology	219	15	35.7	0	138	24.7
	434	6	14.3	230	115	20.6
	542	5	11.9	219	36	6.4
	Others (10)	19	38.1	Others (33)	270	48.3

**Table 3.** Average relative contribution of each environmental variable to the *Lesbia nuna* environmental niche model. C%: percent contribution values; PI: permutation importance; ALL = model combining all variables; CON = model based uniquely on continuous variables (bioclimatic and elevation); C+G = model combining continuous and geologic variables; C+S = models combining continuous and soil variables.

	**ALL**		**CON**		**C+G**		**C+S**	
Variables	C%	PI	C%	PI	C%	PI	C%	PI
Bio03	0.6	2.5	15.5	13.5	3.1	4.7	1.0	1.1
Bio04	0.2	1.3	11.3	14.5	2.2	12.0	0.5	2.3
Bio07	0.1	0.0	0.5	0.8	0.7	1.3	0.2	0.1
Bio12	3.2	3.3	9.3	1.7	4.4	0.8	2.6	8.2
Bio14	2.1	4.1	9.5	2.9	5.0	4.5	2.4	0.5
Bio15	1.6	3.2	5.3	8.3	1.8	6.0	1.8	1.5
Bio18	1.6	7.6	4.4	11.5	1.6	10.2	2.4	13.0
Elevation	19.6	22.1	44.2	46.8	29.4	37.0	20.9	27.9
Geology	14.1	9.3	-	-	51.8	23.5	-	-
Soil	57	46.5	-	-	-	-	68.1	45.3
